# Empirical delineation of the forest-steppe zone is supported by macroclimate

**DOI:** 10.1038/s41598-023-44221-4

**Published:** 2023-10-13

**Authors:** Ákos Bede-Fazekas, Péter Török, László Erdős

**Affiliations:** 1 Institute of Ecology and Botany, HUN-REN Centre for Ecological Research, Alkotmány u. 2-4., 2163 Vácrátót, Hungary; 2 Department of Environmental and Landscape Geography, Faculty of Science, Eötvös Loránd University, Pázmány Péter sétány 1/C., 1117 Budapest, Hungary; 3 HUN-REN-UD Functional and Restoration Ecology Research Group, Egyetem tér 1., 4032 Debrecen, Hungary; 4 Department of Ecology, University of Debrecen, Egyetem tér 1., 4032 Debrecen, Hungary

**Keywords:** Biogeography, Ecological modelling, Forest ecology, Grassland ecology

## Abstract

Eurasian forest-steppes form a 9000-km-long transitional zone between temperate forests and steppes, featuring a complex mosaic of herbaceous and woody habitats. Due to its heterogeneity regarding climate, topography and vegetation, the forest-steppe zone has been divided into several regions. However, a continental-scale empirical delineation of the zone and its regions was missing until recently. Finally, a map has been proposed by Erdős et al. based on floristic composition, physiognomy, relief, and climate. By conducting predictive distribution modeling and hierarchical clustering, here we compared this expert delineation with the solely macroclimate-based predictions and clusters. By assessing the discrepancies, we located the areas where refinement of the delineation or the inclusion of non-macroclimatic predictors should be considered. Also, we identified the most important variables for predicting the existence of the Eurasian forest-steppe zone and its regions. The predicted probability of forest-steppe occurrence showed a very high agreement with the expert delineation. The previous delineation of the West Siberia region was confirmed by our results, while that of the Inner Asia region was the one least confirmed by the macroclimate-based model predictions. The appropriate delineation of the Southeast Europe region from the East Europe region should be refined by further research, and splitting the Far East region into a southern and northern subregion should also be considered. The main macroclimatic predictors of the potential distribution of the zone and its regions were potential evapotranspiration (zone and regions), annual mean temperature (regions), precipitation of driest quarter (regions) and precipitation of warmest quarter (zone), but the importance of climatic variables for prediction showed great variability among the fitted predictive distribution models.

## Introduction

Eurasian forest-steppes extend as a 9000-km-long and on average 400-km-wide belt from the Carpathian Basin (eastern central Europe) to the Amur Lowland (Russian Far East near the Pacific coast) forming a transitional zone between temperate forests and steppes^1^. Forest-steppe is defined as a landscape-scale mosaic of herbaceous and woody habitats in the temperate zone with 10–70% arboreal cover^1^. Due to their complex and mosaic structure, forest-steppes host numerous endemic and/or endangered taxa and have very high diversity at multiple spatial scales^1^. Forest-steppes provide livelihoods^2,3^ and essential ecosystem services^4–6^ for many people. However, vast forest-steppe areas have been turned into arable land or tree plantations and only a small percentage of the forest-steppe area is legally protected^7^.

Even though extremely important both from a theoretical and a practical perspective, the delineation of the forest-steppe zone is rather difficult due to the almost total eradication of natural or near-natural forest-steppe vegetation in several potential forest-steppe regions. Delineation has been done mainly at national, regional, or subcontinental scales (e.g.,^4,8–10^), which makes them unsuitable for studies with a focus on the total forest-steppe zone. On the other hand, most global maps do not distinguish the forest-steppe zone or any equivalent category (e.g.,^11–14^). The first empirical maps of forest-steppe distribution at the continental scale for the whole Eurasian distribution have been published quite recently^1,3^.

The forest-steppe zone covers a vast area and is characterized by marked east–west gradients in terms of climate and vegetation. Accordingly, the zone has been divided into several regions by different authors^1,4,8,9^, indicating that the forest-steppe zone should not be considered climatically homogeneous. However, considerable debates exist regarding the extent of the individual regions (especially in the westernmost and easternmost areas of the forest-steppe zone^3,4,8,15^) and the position of the boundaries between the regions (particularly in the Inner Asian areas^16–18^). Erdős et al.^1^ delineated the Eurasian forest-steppe zone and divided it into regions, following earlier publications and based on floristic composition, physiognomy, relief, and climate.

Currently, it is widely accepted among vegetation ecologists that climate is the primary and most important driver explaining the stable existence and the distribution of forest-steppes (e.g.,^1,3,8,19,20^). However, the existence of a forest-steppe mosaic has been posing an ecological riddle to scientists for the last two hundred years. Various theories were reviewed by Wilhelmy^21^, Chibilyov^8^ and Erdős et al.^22^, and possible explanations include topography, soil salinity, grazing, fire, and anthropogenic forest clearing.

Aridity is able to constrain tree growth^23–25^ and thus may be the most important factor limiting the extension of forests. Apart from special edaphic circumstances (e.g., south-facing steep mountain slopes with shallow soils^4^), the boreal and the temperate deciduous zones are sufficiently humid to support forest vegetation. With increasing aridity towards the south, steppe patches appear amidst forest and become progressively larger while forest patches get smaller^4^. The strong decrease of forest cover, under natural circumstances, towards the south^26^ indicates that aridity, i.e., the ratio of the potential evapotranspiration and the precipitation, limits forest expansion. Similarly, other researchers^4,8,27^ argued that mean annual precipitation and potential evapotranspiration are the most critical parameters. According to Liu^28^, mean annual precipitation is the decisive factor for the emergence of forest-steppes. Moreover, temporal variations and extremes may be as important for the vegetation as long-term means. The forest-steppe zone is characterized by considerable interannual variations (e.g.,^6,8,29^).

Despite the necessity of understanding the distributional limits and main predictors of the forest-steppe zone and its regions, to the best of our knowledge, such analyses have never been conducted at a continental scale. We aim to fill this striking research gap by answering three questions. (1) How do the macroclimate-based predictions relate to the previous empirical delineation and subdivision of the zone by Erdős et al.^1^? (2) Where should refinement of the delineation or the inclusion of non-macroclimatic predictors be considered due to the mismatch of macroclimate-driven analyses and the delineation? (3) What are the most important variables for predicting the existence of the Eurasian forest-steppe zone, and do these variables differ for predicting its regions?

## Results

### Delineation of the forest-steppe zone

A predictive distribution model was successfully trained to distinguish the forest-steppe zone from its surrounding zones (hereinafter 'zone' model). An excellent goodness-of-fit value (AUC = 0.855) was calculated on the evaluation dataset^30^ using the selected macroclimatic variables. When macroclimate was replaced by coordinate-related variables, the goodness-of-fit value was slightly lower (AUC = 0.830). The negligible difference suggested that macroclimate has a strong spatial structure that alone could explain the distribution. The supplementary analysis, i.e., analysis of the shared space-environment fraction (SSEF) by variance partitioning, revealed that 51% of the total variation is explained by the pure spatial effect and the SSEF is 12% (p < 0.01). This suggests that however high the goodness-of-fit values of the predictive distribution models are, only 12% of the found relationship can be surely attributed to the macroclimate itself during the interpretation of the results.

The model simplification step during the predictive distribution modeling suggested the removal of annual precipitation. The relative importance for prediction of the remaining five macroclimatic variables were found to be nearly equal, spanning 13–30%. The two variables characterizing the warmest quarter contributed the most to the predictive distribution model (precipitation of warmest quarter—28.30%; temperature of warmest quarter—24.51%) followed by annual mean temperature (17.55%) and aridity (16.25%). Precipitation of driest quarter showed the lowest contribution (13.39%).

**Figure 1:**
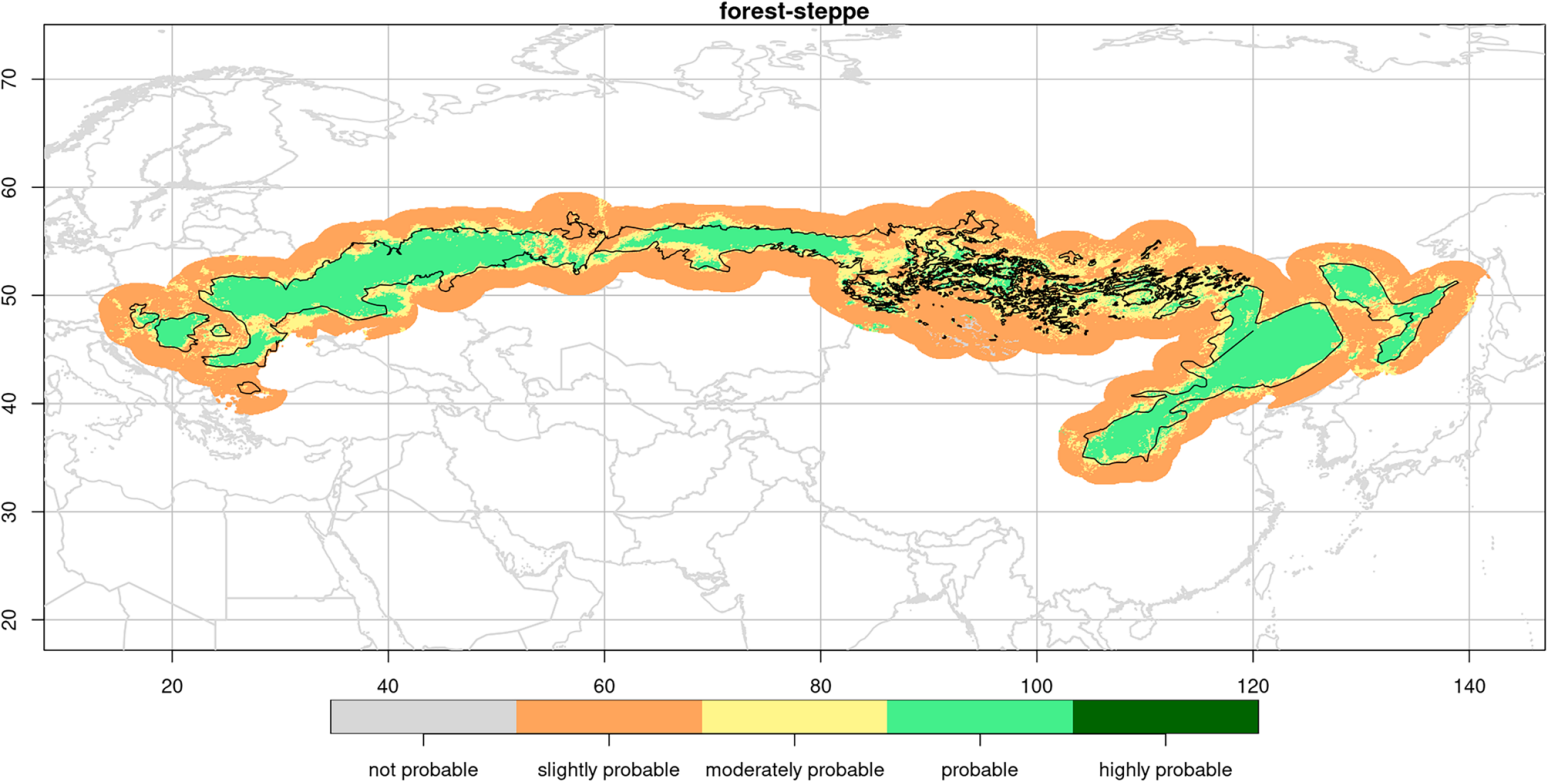
Predicted probability of occurrence of the forest-steppe zone according to the model predicting the potential distribution of the whole zone at the Eurasian level. The distribution delineated by Erdős et al.^1^ is displayed with solid black line.

**Figure 2:**
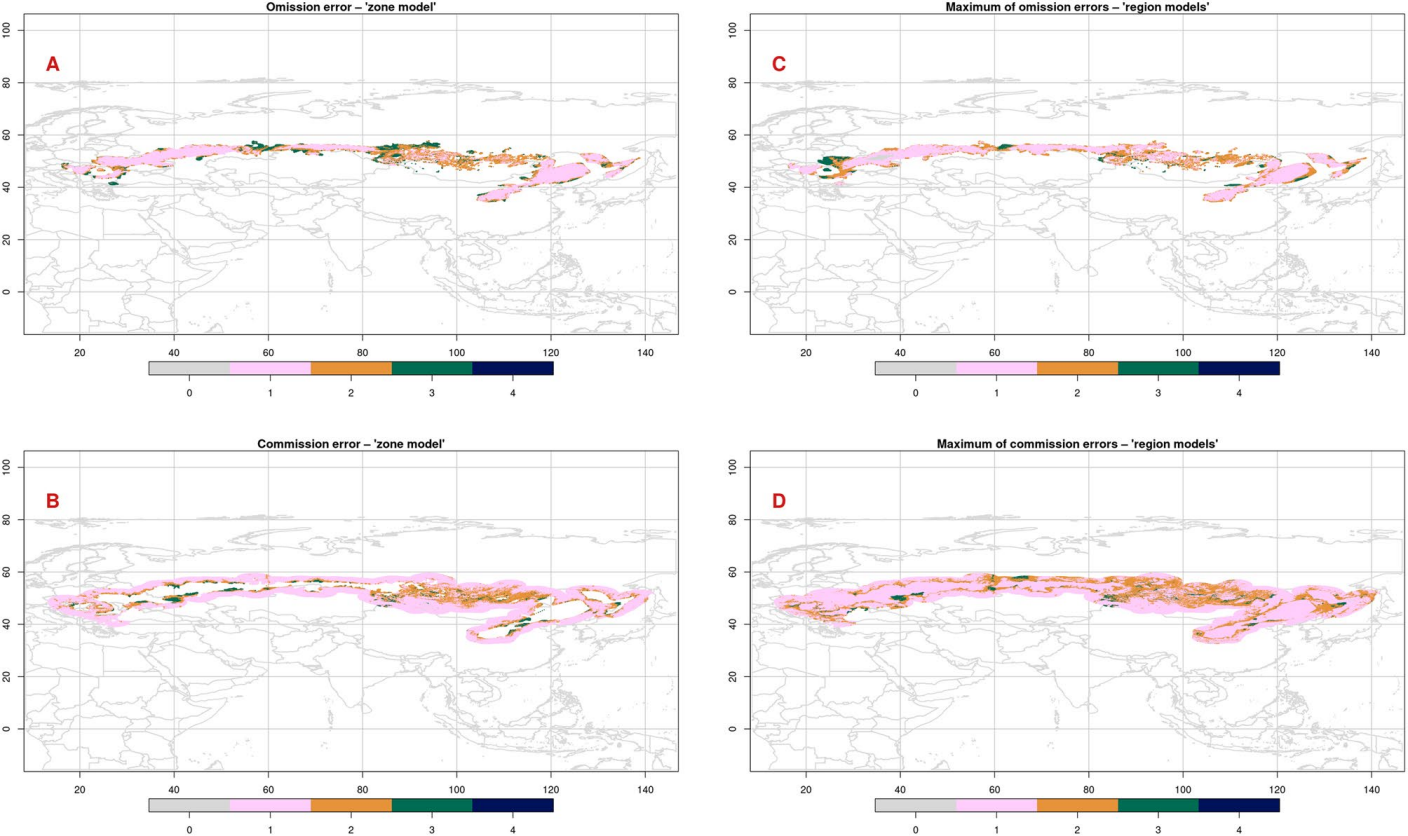
Omission error (**A**) and commission error (**B**) of the 'zone' predictive distribution model, and the aggregation (maximum) of the omission errors (**C**) and commission errors (**D**) of the 'region' predictive distribution models.

The rescaled prediction of the probability of occurrence of the forest-steppe zone (Fig. 1) showed a high degree of agreement with the distribution previously delimited by Erdős et al.^1^. The highest and lowest probability ranks ('highly probable', 'not probable') were rarely predicted. The occurrence of most of the within-zone points were predicted to be 'probable' and some of them to be 'moderately probable'. The occurrence of forest-steppe zone was predicted to be 'slightly probable' for most of the out-of-zone (i.e., non-forest-steppe) points. However, remarkable differences occurred between the observed and predicted distribution, which are summarized in Fig. 2. Most of the within-zone points were underpredicted by one rank (Fig. 2A). Two or three ranks difference occurred mainly in the Inner Asia region and the eastern part of West Siberia region, but the following territories were also prone to underprediction: southern islet of Southeast Europe region in Turkey, southern protrusion of the East Europe region on the right bank of the Volga River, southern border of West Siberia region near northern Kazakhstan, and some northern parts of the Far East region near the Birobidzhan, Russia. The shared boundary of the Southeast Europe and East Europe regions, and that of the East Europe and West Siberia regions near the Ural Mountains were also underpredicted. It is noteworthy that the model was trained on the whole forest-steppe zone, and these underpredictions are independent of the shared boundary suggested by Erdős et al.^1^.

Overpredictions (Fig. 2B) were found mainly near the concave boundary sections of the forest-steppe zone (north of Kyiv, Ukraine; north of Kryvyi Rih, Ukraine; the plains between Sievierodonetsk, Ukraine and Atkarsk, Russia; Ryazan, Russia; north of Buzuluk, Russia; north of Ulanqab, China; south of Datong, China; east of Lyuliang, China), and the most overpredictions occur near the East Europe and Inner Asia regions. Smaller overpredictions occurred also near the Southeast Europe (near Budapest, Hungary) and West Siberia (north of Abatskoye, Russia) regions. Similar to the underpredictions, overpredictions tend to occur near the boundary of the forest-steppe zone, with only a few exceptions near the Inner Asia region.

### Delineation of the forest-steppe regions

According to the preliminary ordination (NMDS) conducted to assist variable selection prior to the predictive distribution modeling, the selected seven macroclimatic variables could reveal remarkable separation of the regions of the forest-steppe zone (Fig. S1.1). The two westernmost regions, i.e., Southeast Europe and East Europe, were isolated from the other three regions, and this isolation is correlated mainly with the annual mean temperature, annual precipitation and the precipitation of driest quarter. Annual mean temperature explained the difference between the two European regions. The West Siberia region, which is situated in the middle of the geographical (i.e., longitudinal) gradient, was placed near the origin of the ordination space. Its little spread (along the annual precipitation gradient) in the ordination space was in contrast to its large geographic extent. The least separated regions were the easternmost ones (i.e., Inner Asia and Far East): the ordination could not clearly distinguish them in two dimensions based on the selected variables. Inner Asia showed the largest variation, suggesting that this region is climatically heterogeneous (in terms of precipitation-related variables). The variation of the Far East region was correlated mainly with isothermality and temperature seasonality, similarly to the West Siberia region. The two-dimensional ordination of the environmental space suggested that the Inner Asia region, which was split into two distinct parts, might be the combination of two subregions disjunct in the geographical space as well. Answering this question is in the scope of hierarchical clustering and predictive distribution modeling. A more detailed description of the ordination along with the relevant figures is presented in Appendix S1.

**Figure 3:**
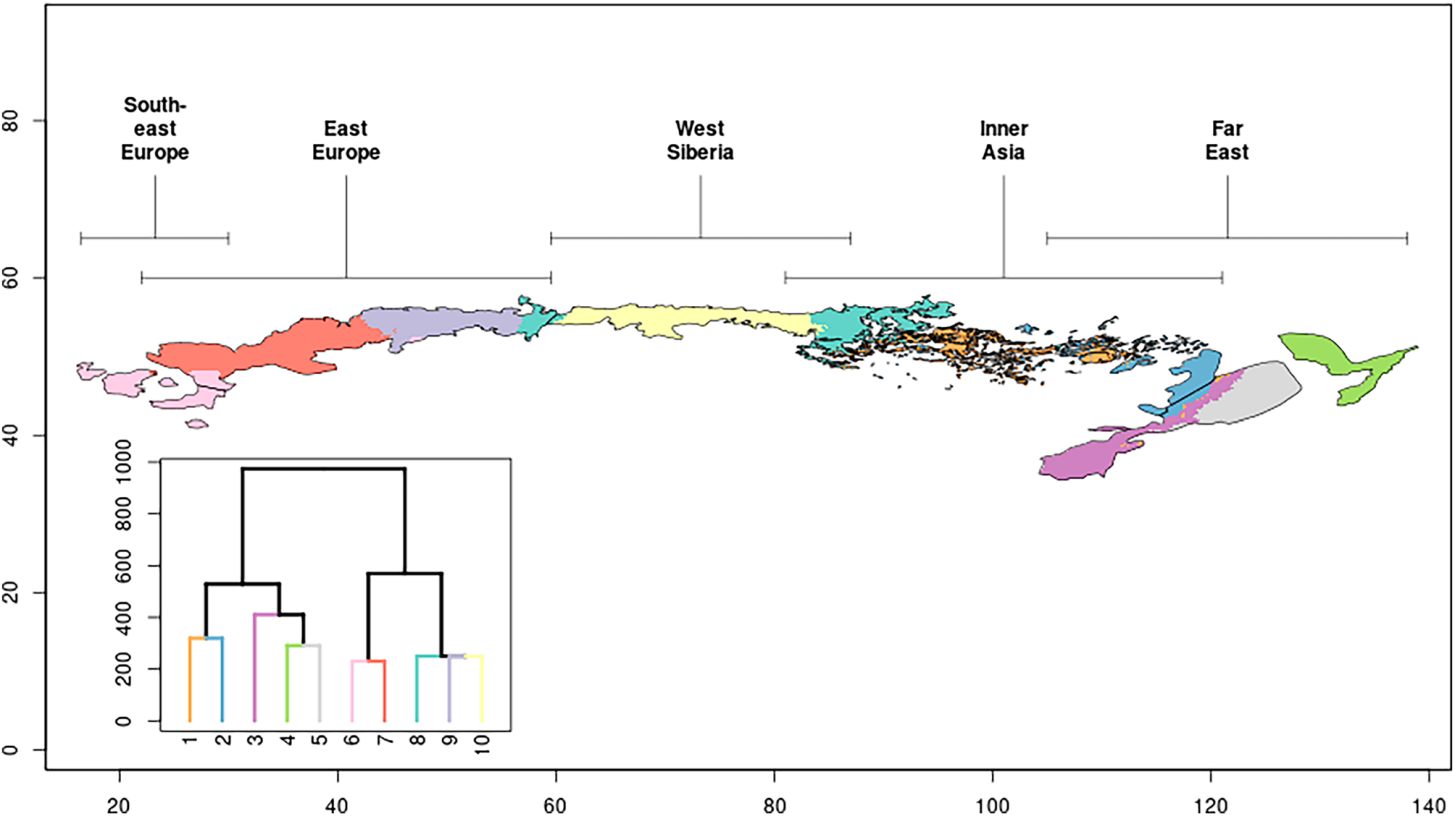
Result of the hierarchical clustering of the forest-steppe zone into ten clusters (bottom left subfigure) achieved on all macroclimatic variables, and the distribution of these clusters in the geographic space (main figure). The colors of the main figure and the subfigure match. Regions originally delineated by Erdős et al.^1^ but not used as input by the clustering are labeled and displayed with solid black lines for comparison purposes.

Hierarchical clustering was used to form clusters of the macroclimatic space and display them in the geographic space without prior information on the delineation of the regions by Erdős et al.^1^ to explore whether this delineation of the regions is in agreement with the climate-driven clusters. In the ten-cluster resolution hierarchical clustering (Fig. 3), the forest-steppe zone was separated firstly into a western (no. 6–10) and an eastern (1–5) main cluster. The western main cluster consists of Southeast Europe, East Europe, West Siberia, and some isolated parts of Inner Asia. Near the foothills of the Altai Mountains, the border of the western and eastern central clusters gives strong support to the previously drawn demarcation line between the West Siberia region and the Inner Asia region. However, the climate of the northern protrusion of the Inner Asia region towards the West Siberian Plain (east of the Kuznetsk Alatau Mountains) was found to be similar to that of the three western regions. Then the western main cluster was divided into a western (6–7) and a eastern part (8–10) at the Atkarsk, Russia–Kasimov, Russia line. Then the eastern main cluster was separated into a western part (1–2) formed mainly by the Inner Asia region and an eastern part (3–5) formed mainly by the Far East region. The division clearly distinguished the climate of the Inner Asia and Far East regions, but the hierarchical clustering suggested that the southern island of the Inner Asia region (north of the Yellow River) may belong to the Far East region, while the northern shared boundary of the two regions might be shifted eastward. In the next steps, the eastern main cluster was further separated into five clusters, among which cluster 4 matched exactly the northern part of the Far East region. Then clusters 8, 9 and 10 were separated in a way that partly confirmed the delimitation of East Europe and West Siberia region near the Ural Mountains as suggested by Erdős et al. previously^1^. However, the results of the hierarchical clustering (i.e., the disjunct nature of cluster 8) revealed the climatic heterogeneity of the West Siberia region along a longitudinal gradient. Finally, clusters 1 and 2 were separated near the Moldavian–Ukrainian border suggesting the refinement of the division of the Southeast Europe and East Europe regions. The further subdivision of the ten clusters was not found worthy of interpretation.

Although the selected clustering algorithm was not geographically constrained (in contrast to e.g., SKATER algorithm), the resulting clusters are mostly contiguous in the geographical space (Fig. 3). This suggests that the selected macroclimatic variables well describe the geographical distances by their Euclidean distances and that the geographically delimited regions are climatically homogeneous if compared to the climatic heterogeneity of the whole forest-steppe zone.

All the five predictive distribution models trained on the forest-steppe regions separately (hereinafter 'region' models) reached an AUC value above 0.9 (mean = 0.958; sd = 0.022; Table 1). According to this measure, the 'region' predictive distribution models performed better than the 'zone' model, which is presumably caused by the smaller geographic extent of the regions with less climatic heterogeneity. When macroclimate was replaced by coordinate-related variables, the AUC values mostly decreased (mean = 0.900, sd = 0.009), but slight increases also occurred (East Europe and Inner Asia regions). The small and uncertain difference let us recall our previous warning on the limitations of interpreting macroclimate as the sole driver of the distribution of the forest-steppe.

**Table 1:** Relative importance for prediction (%) of the macroclimatic variables, goodness-of-fit value (AUC) and the goodness-of-fit value ('AUC with coordinates') if macroclimate is replaced by coordinate-related variables for all the five 'region' models. Variables with high contribution (> 20%) are bolded.

Region	Aridity	Annual mean temperature	Isothermality	Temperature seasonality	Annual precipitation	Precipitation of driest quarter	Precipitation of warmest quarter	AUC	AUC with coordinates
Southeast Europe	2.77	**38.20**	–	**22.27**	3.63	**27.34**	5.79	0.979	0.726
East Europe	**21.55**	7.92	14.84	10.15	–	**41.06**	4.49	0.953	0.981
West Siberia	**26.71**	**28.95**	3.75	5.55	–	**32.14**	2.89	0.953	0.913
Inner Asia	13.60	**30.22**	10.85	13.99	–	11.75	19.60	0.922	0.929
Far East	18.07	**22.13**	–	13.04	17.22	15.35	14.19	0.981	0.953

Further details (i.e., learning rate and number of trees) can be found in Table S2.1.

All the five 'region' models dropped one variable (Table 1). Isothermality and annual precipitation were dropped by two and three models, respectively. The other four variables were found important for prediction by each model, among which annual mean temperature, precipitation of driest quarter and aridity contributed the most to the models. Annual mean temperature occurred most frequently among the variables most important for prediction (i.e., > 20% contribution). If studied model-wise, the variables with the highest contribution were annual mean temperature (Southeast Europe, Inner Asia, Far East) and precipitation of driest quarter (East Europe, West Siberia). For each of the 'region' models, relative importance for prediction of the variables showed larger variation than in the case of the predictive distribution model trained on the whole forest-steppe zone. This suggests that the distribution of the regions can be more firmly defined by macroclimate than the whole zone, which is in agreement with the higher goodness-of-fit values of these 'region' models.

Rescaled predictions with the 'region' predictive distribution models are shown in Figs. S3.1–3.5. The maps suggested that all five models could roughly corroborate the delimitation of the studied region, but both over- and underpredictions may occur at fine scale. The least specific models were those of the Inner Asia and Far East regions: they predicted hardly any 'not probable' and 'highly probable' areas. The model of the East Europe region was the most sure about the presences (i.e., predicted the most 'highly probable' points), while the models of West Siberia and Southeast Europe regions were the most sure about the absences according to the number of 'not probable' points. However, this latter model was not sure about the middle island of the region (Transylvania, Romania) and predicted that occurrences in some southern territories near the Far East region are 'moderately probable', suggesting climatic similarities between these remote parts of Eurasia. Also, the models of West Siberia (towards west and north) and Inner Asia (towards north and east) regions made remarkable overpredictions (commission errors).

Aggregated omission (Fig. 2C) and commission (Fig. 2D) errors suggested that the delimitation of the zone and regions may be revised near the Carpathians, south of the East Europe region, the Ural Mountains, north of the West Siberia region, the Altai Mountains and the area between the southwestern and northeastern part of Far East region. This latter finding is in contrast with the clear separation suggested by the cluster analysis. Regarding the omission errors, the East Europe region gained the most accuracy by being modeled separately: its 'region' model showed much lower omission errors. If the maximum of commission errors was studied, border of the East Europe and West Siberia regions (near the Ural Mountains) and the surroundings of the Inner Asia region seemed to show the greatest similarity to one or more other regions, which makes the separation of these regions from the others more difficult.

Patterns found by the aggregated omission (Fig. 2C) and commission (Fig. 2D) errors showed similarities to those of the errors of the 'zone' model (Fig. 2A,B), but differences also occurred. For example, the southern islet of the Southeast Europe region in Turkey (presence), the eastern part of the East Europe region (presence), north of the East Europe region (absence) and west of the Kuznetsk Alatau Mountains (presence) were well predicted by the 'region' models in contrast to the 'zone' model. However, the 'zone' model was not always overperformed by the 'region' models: remarkable omission errors (e.g. Transylvania and West Ukraine) and commission errors (e.g. northern part of the Far East region) were also made by the 'region' models.

## Discussion

### Delineation of the forest-steppe zone

Our results showed that the forest-steppe versus non-forest-steppe separation is not sharp (Figs. 1, S3.1–3.5), suggesting that the biome boundaries under study (i.e., the boundaries of the forest-steppe towards the adjacent biomes) are gradual. This is in line with earlier observations regarding how forest-steppe transitions into the neighboring vegetation zones (e.g.,^1,8^), and, at a more fundamental level, also reflects the usually continuous nature of spatial environmental gradients (e.g.,^31^).

Both the ordination and the estimation of variables' importance for prediction by the predictive distribution models emphasize that the two variables mostly related to drought, i.e., precipitation of driest quarter and aridity, are powerful predictors of forest-steppe formation. This is in line with earlier opinions on the importance of this factor (e.g.,^4,6^). Some of the annual variables, i.e., annual precipitation, isothermality and temperature seasonality, seem to have a weaker predictive power. Growing season factors (i.e., temperature of warmest quarter, precipitation of warmest quarter and precipitation of driest quarter), which may play an important role in limiting tree survival and thus hindering the formation of closed forests, had a remarkable contribution to the predictive models.

According to the 'zone' predictive distribution model, the probability of forest-steppe occurrence showed a very high agreement with the forest-steppe zone delineation of Erdős et al.^1^ used for the model training (Fig. 1), which is in line with the excellent goodness-of-fit value of the model. Since the model was trained on macroclimatic variables, the results might impel the excessive interpretation that the forest-steppe zone is under a strong macroclimatic control. However, supplementary analysis of the shared space-environment fraction and the goodness-of-fit of models trained on coordinate-related variables instead of macroclimate warn that the distribution pattern of the forests-steppe zone could not be explained only by the selected macroclimatic variables. Therefore, finer resolution future studies that are more focused on a selected part of the zone are needed to refine our findings by expanding the set of predictors with those more relevant at regional scale (e.g., edaphic parameters, groundwater availability, and topographic heterogeneity).

Process-based vegetation models usually do not regard forest-steppe as a separate zone but consider it to belong either to the forest or the steppe zone (e.g.,^32–34^). Process-based models that treat forest-steppe as a separate zone typically cannot correctly reproduce this zone (e.g.,^35,36^). One possible reason for this poor performance may be that transitional zones are difficult to model^37^. A better understanding of the predictors of the coarse-scale distribution of forest-steppe can support the better parametrization of process-based models and thus may contribute to more realistic predictions.

In continental-scale macroclimate-based analyses of distributions, the spatial resolution of the distribution needs to be as fine as possible to match the currently available fine-resolution climate datasets. Resolution mismatch might result in findings not well established. Although we incorporated all the available regional maps during the refinement of the forest-steppe delineation, we suggest using more maps for further refinement, e.g. the map of Ogureeva et al.^38^ and Samoylova^39^.

### Delineation of the forest-steppe regions

Some of the findings of the ordination (Fig. S1.1) can be well explained by previous knowledge of these forest-steppe regions. The small climatic variation of the Southeast Europe region is at least partly due to its relatively small geographical extent. However, the compact mapping of the West Siberia region in the ordination space indicates that this region is, in contrast to its large longitudinal extension, climatically rather homogeneous. The large variation of the Far East region along the change of temperature seasonality and isothermality may be explained by the large altitudinal range of forest-steppes (50–2500 m a.s.l.^1^). In addition, there is a steep aridity gradient from the Pacific Ocean to the inner areas of the continent^40,41^, resulting in large climatic differences over small spatial distances. The largest variation arose within the Inner Asia region, probably reflecting the variable conditions under which forest-steppes are found in this region, from low valleys to high mountains^27,42^.

The fact that climatic predictors differ among the main forest-steppe regions emphasizes that several factors should be considered when explaining why a certain area supports a forest-steppe mosaic. For example, in some areas of the Southeast Europe region, mean annual precipitation could be enough to support forest vegetation, but the drought period in summer may hinder the establishment of tree seedlings and thus can contribute to the existence of a forest-steppe mosaic^43–45^. Similarly, where annual precipitation is relatively high (Southeast Europe region and parts of the East Europe region, Fig. S1.1), natural (i.e., pre-human) wildfires and herbivores may have played a decisive role in limiting forest vegetation and maintaining the forest-steppe mosaic. Fire and grazing were probably especially important in limiting forests at the northern and western fringes of the forest-steppe zone (cf.^22,44,46,47^). In contrast to areas where summer rain is typical, fires may be more frequent and more intensive where the precipitation of the warmest quarter is low, such as in the Southeast Europe region as well as in some parts of the Inner Asia and Far East regions (see Fig. S1.1). Wildfires played an important role in these regions and are believed to have contributed to the prevention of forest canopy closure (e.g.,^44,46,48^).

The ordination revealed a clear separation of Southeast and East Europe regions from the other ones along the gradient of the precipitation of driest quarter, which is relatively large in these two regions but tends to decrease towards the east (Fig. S1.1). This has a marked effect on the vegetational differences among the regions. For example, precipitation during the winter and early spring supports geophytes, which can flourish from Southeast Europe to West Siberia but play a subordinate role in the Inner Asia region, where winters and springs are very dry^49^. This again underlines that seasonal climate values may in some cases be ecologically more important than annual means.

The ten clusters defined by the hierarchical clustering in the present study (Fig. 3) showed a good overall agreement with the regions previously identified by Erdős et al.^1^. The results of the macroclimate-based clustering, which was independent of the previous delineation, confirmed the separation of four out of the five regions. However, considerable differences did arise that suggest the revision of the delineations in some cases. Distributions of the regions might partly be explained by non-climatic factors such as herbivory and fire, which inevitably contribute to the mismatch. The western part of the East Europe region seems to be climatically close to the Southeast Europe region. This probably reflects Sub-Mediterranean climatic influences from the Balkan Peninsula, which can proceed unhindered towards the north and northeast. As a result, Sub-Mediterranean vegetation is present in small patches along the eastern foothills of the Carpathian Mountains^50^. Nevertheless, from north Moldova onwards the vegetation is more and more continental^50,51^ and thus we think the original boundary delineated by Erdős et al.^1^ is defensible.

According to our analysis, the East Europe region is split into two parts. Earlier works on the forest-steppe vegetation of the region subdivided it into smaller units differently: while Walter and Breckle^4^ and Lavrenko and Karamysheva^9^ identified two inner boundaries, the map of Chibilyov^8^ shows six inner boundaries. However, none of these correspond to the boundary suggested by our climatic analysis.

The boundary between the East Europe and the West Siberia regions identified by the present analysis coincides well with the boundary described by earlier works^1,4,8,9,52^. Due to their north–south direction, the Ural Mountains present a considerable obstacle to the westerly winds, which results in increased continentality, both in terms of climate and vegetation.

The Inner Asia region was found to be climatically heterogeneous. The areas north of the Altai Mountains (the northern protrusion of the Inner Asia region towards the West Siberian Plain) were confirmed to be climatically closely related to the West Siberia region. The position of the boundary between the West Siberia and the Inner Asia region has long been a subject of scientific debate (e.g.,^9,16,18,53^). Climatic influences from the West Siberian Plain are able to reach the area north of the Altai Mountains, which results in floristic similarity between the two areas^54^. In light of these results and contrary to Erdős et al.^1^, the area in question should probably be regarded as belonging to the West Siberia region.

The boundary between the Inner Asia and the Far East regions coincides well with the boundary delineated by Erdős et al.^1^. Only a ca. 100 km southeastward shift is suggested by the clustering that would separate the Inner Asia region and the Far East region at lower altitude. However, the southern island of the Inner Asia region (north of the Yellow River) might be assigned to the Far East region in the future. The climatic classification split the Far East region into three parts. Among them, the northeastern cluster exactly coincides with the spatially distinct northeastern polygon of the region suggesting that the geographical separation was echoed by the climate-driven clustering. Erdős et al.^1^ treated the Far East region as one unit partly because of the strong floristic similarity between Manchuria (within the southwestern polygon) and the Russian Far East (the northeastern polygon)^55,56^. Also, there are notable faunistic similarities between the two polygons^57^. Nevertheless, Erdős et al.^1^ already noted the marked climatic differences between the more continental southwestern and the cooler northeastern parts of the Far East region. In fact, the northeastern part is so humid and cool that it is debated whether or not it belongs to the forest-steppe zone. For example, Tishkov et al.^58^ treat the area as part of the temperate forest zone, Zlotin^5^ as part of the forest-steppe zone, while Wesche et al.^3^ think that small areas of the polygon belong to the forest-steppe and the rest to the temperate forest zone. Based on an analysis of climatic conditions, Novakovsky^59^ concluded that forest-steppe is the natural vegetation only on dry mountain slopes, whereas closed forests are natural elsewhere. Darman et al.^60^ argue that fires played here an important role in preventing the closure of the forest canopy and thereby maintaining forest-steppe vegetation. Our results in the present work emphasize the climatic differences between the northeastern part of the Far East region and the rest of the region.

The predicted probability of occurrences of the individual regions according to the predictive distribution models (Figs. S3.1–3.5) showed a good overall agreement with the forest-steppe regions of Erdős et al.^1^, but the predicted probability of occurrence of the Inner Asia region (Fig. S3.4) also highlighted the topographic and climatic heterogeneity of this region^42^. Similarly, the aggregated omission and commission errors (Fig. 2C,D) of the 'region' predictive distribution models also suggest that the delineation done by Erdős et al.^1^ needs further refinement or non-macroclimatic predictors should also be considered in these areas.

The predictive distribution models agreed also with some findings of the cluster analysis. For example, the hierarchical clustering suggested the reconsideration of the southern island of the Inner Asia region (north of the Yellow River), which was also confirmed by the low predicted probability of occurrence (i.e., high omission error) of the model of this region (Figs. 2C, S3.4). An interesting contradiction was, however, that both the East Europe and Far East regions were separated sharply into two parts by the cluster analysis (clusters 7 vs. 9, and 4 vs. 5, respectively), while the 'region' models made low omission error within the East Europe region (Fig. 2C) and high commission error between the southwestern and northeastern part of the Far East region (Lesser Khingan Mountains, Fig. 2D).

## Conclusions

The predictive distribution models of the forest-steppe zone and its regions with excellent goodness-of-fit values clearly corroborated that the large-scale distribution can be modeled by using the selected macroclimatic variables. Aridity, annual mean temperature and precipitation of driest quarter seem to be the most important predictors of the regions and variables describing the warmest quarter showed the highest contribution to the model of the zone when separation from the surrounding areas was studied. Supplementary analyses, however, suggest that the delimitation of the forest-steppe zone from its surroundings can only partly be attributed to the selected macroclimatic variables.

The preparatory ordination analysis revealed that macroclimate can partly describe the delimitation of the five forest-steppe regions. The hierarchical cluster analysis of the macroclimatic variables, which was independent of the previous delineation, found that macroclimate on its own can support the distinction of most of the forest-steppe regions. Over- and underpredictions of the predictive distribution models, in agreement with the results of the hierarchical clustering, suggest that the boundary between the East Europe and West Siberia regions and also between the West Siberia and Inner Asia regions may benefit from small-scale refinement. The clustering agreed with the ordination in that the Far East region should be subdivided. A ca. 100 km southeastward shift of the shared border of Inner Asia and Far East regions is suggested by the clustering. The appropriate delineation of the Southeast Europe region from the East Europe region needs further research.

Detailed analyses of the forest-steppe biome should not treat the whole zone as one unit, as this may mask the considerable differences among the regions. The macroclimate-distribution relationship is easier to be characterized from the regional-scale analysis than from the biom-scale analysis.

## Material and methods

### Research framework

A large variety of statistical methods is suitable for studying the delineation and subdivision of a spatial unit, such as a biome, that may be driven by macroclimate (e.g.^13,61–65^). We selected two approaches complementing each other to answer our three research questions: predictive distribution modeling and hierarchical clustering. (1) Prediction made by the distribution model, evaluation of the model and the study of the clusters produced by the hierarchical clustering answer whether macroclimate can predict the previous empirical delineation and subdivision of the zone. (2) The areas where refinement of the previous delineation or the inclusion of non-microclimatic predictors is suggested can be located by exploring the potential inaccuracies (i.e., under and overestimations) of the distribution model and the mismatch between the delineation of the forest-steppe regions and the cluster boundaries. (3) The main macroclimatic predictors of the forest-steppe zone and its regions can be detected by the variable importance estimation by the predictive distribution model. Details are given in the next subchapters.

### Distribution of the forest-steppe zone and its regions

In the present study we used the authoritative expert map of the forest-steppe zone and its regions compiled by Erdős et al.^1^ as input for the analyses and to compare it with our climate-based prediction. Extrazonal occurrences of forest-steppe (i.e., small patches of forest-steppes found outside the forest-steppe zone, defined by local circumstances such as steep southern slopes or thin soil), which would otherwise increase the uncertainty of our macroclimate-focused analyses, were not included in the map of Erdős et al.^1^ and were not considered by the present study, either. More details on the map of Erdős et al.^1^ can be found in Appendix S4. The map of Erdős et al.^1^ was modified relying primarily on the map of Isachenko^66^ but also consulting Tchebakova et al.^67^, Suvorov et al.^68^ and Olson et al.^69^. Considerable refinements were carried out in the Inner Asia region, while only minor adjustments were made in the other regions. Areas where the forest-steppe character is debated were treated as part of the forest-steppe zone, potentially resulting in local overestimations of forest-steppe occurrence. The revised map is provided in Appendix S5.

Erdős et al.^1^ adopted a broad forest-steppe definition, which includes a northern belt (i.e., forest-steppe zone sensu stricto^4^) and a southern belt of the forest-steppe zone sensu lato in Eurasia. While the northern zone covers a wide latitudinal band between forests and steppes, the southern zone occupies an altitudinal belt in the mountains of the arid regions of the Middle East and Central Asia. The latter is climatically, structurally and compositionally rather distinct from the northern zone, and it is usually only a relatively narrow transitional belt on mountain slopes. The southern zone is less suitable for continental-scale, coarse-resolution analyses since i) its distribution is limited mainly by fine-scale mesoclimate instead of macroclimate^1^, and ii) is under-studied, hence its delimitation is highly uncertain. Therefore, in this study, we focused our attention on the northern (latitudinal) belt of the forest-steppe zone.

### Climatic data and variable selection for the predictive distribution models

Macroclimatic data were obtained from the WorldClim 2.0 database^70^ at 5 min (~ 10 km) horizontal resolution and the Global Aridity Index and Potential Evapo-Transpiration (ET0) Climate Database^71^ at 30 s (~ 1 km) horizontal resolution. The latter was aggregated to 5 min resolution by averaging. Please note that the original weather data served as input for these databases had ≥ 50 km resolution for temperature and ≥ 25 km for precipitation that was downscaled by thin-plate spline interpolator^70,71^. The macroclimate of the 1970–2000 period was described by the 19 bioclimatic variables (^72^, Appendix S6) and aridity (i.e. the ratio of potential evapotranspiration and annual precipitation) that are considered to have more ecological relevance than the raw, monthly climatic data^73,74^. These variables are widely used in large-scale biogeographical studies and predictive distribution models^75–77^.

For the predictive distribution models, we created two subsets of the 20 macroclimatic variables by a statistically and ecologically informed variable selection process. Variable selection improves the transferability of predictive distribution models and is indispensable if the trained models are later used for extrapolation^78–80^. The selection was assisted by the calculation of the correlation matrix of the 20 macroclimatic variables (Appendix S7) and ordination by non-metric multidimensional scaling (NMDS). Due to its preparatory nature, the methodological details and the results of the ordination are provided as supplementary information (Appendix S1).

Both of the variable subsets had to fulfill our multicollinearity criteria: pairwise Pearson's correlation coefficients were limited to |r|< 0.8, Variance Inflation Factor (VIF,^81^) of the variables were maximized at 20, and Condition Number (CN, ^82^) of the variable set was maximized at 10^78^. During the variable selection process, we relied on the statements of scientific literature^4,28^ and the opinion of experts (Oleg Anenkhonov, Anna Kuzemko, Hongyan Liu, Victor Onyshchenko and Yuri A. Semenishchenkov, personal communication) on the relevance of the different variables for stable forest-steppe coexistence.

For the analyses studying the whole Eurasian forest-steppe zone and its demarcation from the surrounding areas (i.e., 'zone' model), we tried to identify those factors that can be assumed to be primarily responsible for the existence of the forest-steppe zone (i.e., factors that likely determine the outcome of the competition of forests and steppes). Therefore, the following macroclimatic variables were selected for the 'zone' subset: aridity, annual mean temperature, mean temperature of warmest quarter, annual precipitation, precipitation of driest quarter and precipitation of warmest quarter.

For the analyses studying the regions separately (i.e., 'region' models), the mean temperature of warmest quarter, which shows little longitudinal variation, was replaced with isothermality and temperature seasonality. The latter variables are assumed to describe continentality and can better explain the separation of the regions.

Although the variable subset for the 'zone' model contained the most highly correlated variable pair (annual mean temperature and mean temperature of warmest quarter), it was less multicollinear (max(|r|) = 0.79; max(VIF) = 14.48; CN = 8.35) than the subset for the 'region' models (max(|r|) = 0.68; max(VIF) = 14.68; CN = 9.16).

### Analyses

In all the analyses, the 5 min resolution rasters were overlapped with the distribution polygons of the zone/regions and the extracted cell values were used as environmental data that describe the cells' centroid.

Hierarchical clustering was used to form clusters of the macroclimatic space using the 20 macroclimatic variables and display them in the geographic space without prior information on the delineation of the regions by Erdős et al.^1^. The climate-driven clusters were then compared to the prior delineation to explore whether this delineation of the regions is in agreement with the climate-driven clusters, and if not, where the independent clustering suggests the refinement of the delineation by Erdős et al.^1^. Euclidean distance matrix of the standardized climatic space between geographical point pairs was clustered using Ward's^83^ agglomeration method.

Predictive distribution models are widely used to predict the potential distribution of species ('species distribution models') but are often applied on other taxonomical or syntaxonomical levels, such as habitats or biomes (e.g.^84–87^). Predictive distribution modeling is the method of finding the relation between the environment and the observed distribution of the studied entity (i.e. training the model) and estimation of the potential distribution (i.e. prediction).

Boosted Regression Trees (BRT) was selected from the many available distribution modeling methods. During predictive distribution modeling, distribution was treated as presence-absence data. For the 'zone' model, we defined the non-forest-steppe area for the statistical comparisons as a 200-km-wide buffer around forest-steppe zone in Asia Lambert Conformal Conic projection (ESRI: 102012). This resulted in a zone whose area (7,196,069 km^2^) is comparable to that of the forest-steppe zone (3,451,016 km^2^). For the 'region' models, points falling within the studied region were used as presences, while both the above-defined buffer and the points falling within the four other regions were treated as absences.

For the six studied distributions, i.e. that of the zone and the five regions, independent models were trained and later used to predict the probability of potential occurrence of the zone and its regions. Predictions, falling within the [0; 1] interval, were rescaled to a five-level ordinal scale (from 'not probable' to 'highly probable') using specific thresholds that account for observed presences^88^. Further methodological details are provided as supplementary information (Appendix S2).

To avoid excessive interpretation of the macroclimatic variables found to be important predictors by the distribution models, the analysis of the shared space-environment fraction (SSEF) by variance partitioning was conducted following the guideline provided by Bauman et al.^89^. This supplementary analysis assisted us in identifying the level we are empowered to interpret the macroclimate as direct predictor of the distribution instead of being a proxy for other, spatially correlated predictors. The significance criteria suggested by Bauman et al.^89^ were checked before variation partitioning. For computational reasons, a sample of size n = 2500 was drawn from the whole 'zone' dataset and the significance of the spatial structure of the presence-absence data and that of the macroclimatic space were determined by using 200 permutations.

All the analyses were conducted in R statistical environment^90^ using packages 'adespatial'^91^, 'blockCV'^92^, 'corrplot'^93^, 'dismo'^94^, 'fastcluster'^95^, 'fasterize'^96^, 'gbm'^97^, 'raster'^98^, 'ROCR'^99^, 'sf'^100^, 'sp'^101,102^, 'usdm'^103^ and 'vegan'^104^.

### Supplementary Information

Supplementary Information S1.

Supplementary Information S2.

Supplementary Information S3.

Supplementary Information S4.

Supplementary Information S5.

Supplementary Information S6.

Supplementary Information S7.

## Data Availability

Climatic data used in the current study are available in the WorldClim 2.0 database http://www.worldclim.org, and the Global Aridity Index and Potential Evapo-Transpiration Climate Database, figshare.com/articles/dataset/Global_Aridity_Index_and_Potential_Evapotranspiration_ET0_Climate_Database_v2/7504448. The distribution of Eurasian forest steppe zone and its regions used as input for our research is provided as a supplementary material in GIS and image formats.
