# Supplementary material for: Empirical delineation of the forest-steppe zone is supported by macroclimate
Source: Sci Rep. 2023 Oct 13;13:17379. doi: 10.1038/s41598-023-44221-4 (PMC10575856; doi:10.1038/s41598-023-44221-4)
Supplement: Supplementary file 1 — Supplementary Information S1. [file 41598_2023_44221_MOESM1_ESM.docx]

# Appendix S1 – Details of the non-metric multidimensional scaling

Ákos Bede-Fazekas et al.: Empirical delineation of the forest-steppe zone is supported by macroclimate. Scientific Reports

## Methods

Non-metric multidimensional scaling (NMDS) was applied as preparatory analysis to (1) assist variable selection for the predictive distribution models and to (2) study whether the regions are separated from each other in the two-dimensional ordination space drawn by the selected macroclimatic variables. This multivariate ordination method tries to place the data points in the ordination space in a way that the rank of the distances between the points in the low-dimensional ordination space best fit the rank of their distances in the high-dimensional environmental (i.e., macroclimatic) space. The level of distortion, i.e., the inability of the algorithm to reach a good fit, was measured with stress value (Kruskal 1964). The NMDS was calculated using the Bray-Curtis dissimilarity matrix (Bray and Curtis 1957) of the standardized data. Due to computational limits, a stratified random sample containing n = 10,000 points from the original dataset was analyzed. Since NMDS is an iterative algorithm, to facilitate convergence to a non-local optimum, several (minimum 20, maximum 1000) NMDSs were run from random starts. Results were visualized using centering and rotation to the axes of principal components analysis. Displaying the macroclimatic variables as directions along with the points (i.e. biplot) was preceded by a thorough study of the Shepard diagram and independent trend surfaces of the variables drawn on the ordination space and was accepted if the ordination space was found to be near metric. During visualization, ordination ellipses were drawn based on the standard deviation of point scores, where weighted correlation defined the direction of the major axis of the ellipse.

## Results

The NMDS ordination of the 20 macroclimatic variables found by the iterative algorithm had a stress value of 0.115. The variables formed groups (Fig. S1.1) that were in accordance with the correlation between the variable pairs (Appendix S7). The variable groups were arranged in different directions of the ordination space, suggesting that candidate variables selected from these groups might be successfully used as predictors in the subsequent predictive distribution modeling step. The NMDS using the seven macroclimatic variables selected according to the variable selection procedure (described in the main text) resulted in an acceptable (i.e., <0.2) stress value of 0.165. The Shepard plot (Fig. S1.2) reached an R^2^ = 0.870 linear fit. The trend surfaces of the macroclimatic variables (Fig. S1.3) confirmed that some variables (annual mean temperature, isothermality, precipitation seasonality, precipitation of driest quarter) can be interpreted as directions. The resulting ordination (Fig. S1.1) showed that (1) the selected seven variables are arranged in different directions of the ordination space; (2) most of them are highly correlated to the two-dimensional ordination space (precipitation of the warmest quarter is the least correlated); and (3) most of the forest-steppe regions except Inner Asia region are separated well in the ordination space by the seven variables.


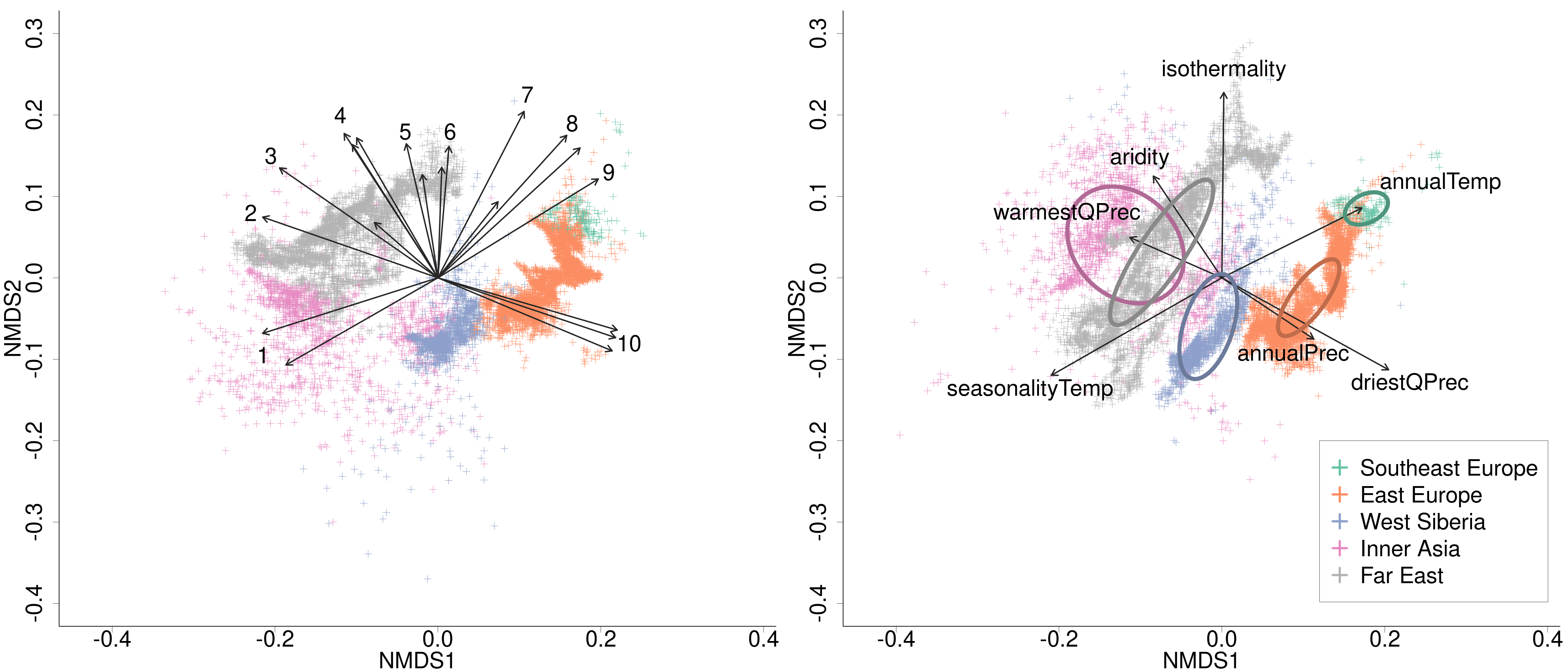
Figure S1.1. Non-metric multidimensional scaling of the forest-steppe regions using all the 20 macroclimatic variables (left panel) and the selected seven variables (right panel). In the 20-variables subfigure, arrows of the variables are grouped and numbered to improve readability (1 – temperature annual range, temperature seasonality; 2 – mean diurnal range; 3 – precipitation seasonality, aridity; 4 – precipitation of wettest month, precipitation of wettest quarter, precipitation of warmest quarter; 5 – isothermality, maximum temperature of warmest month; 6 – mean temperature of wettest quarter, mean temperature of warmest quarter; 7 – annual mean temperature; 8 – annual precipitation, minimum temperature of coldest month, mean temperature of coldest quarter; 9 – mean temperature of driest quarter; 10 – precipitation of driest month, precipitation of driest quarter, precipitation of coldest quarter). Abbreviation applied on the right subfigure are as follows: driestQPrec – precipitation of driest quarter; annualPrec – annual precipitation; annualTemp – annual mean temperature; warmestQPrec – precipitation of warmest quarter; seasonalityTemp – temperature seasonality


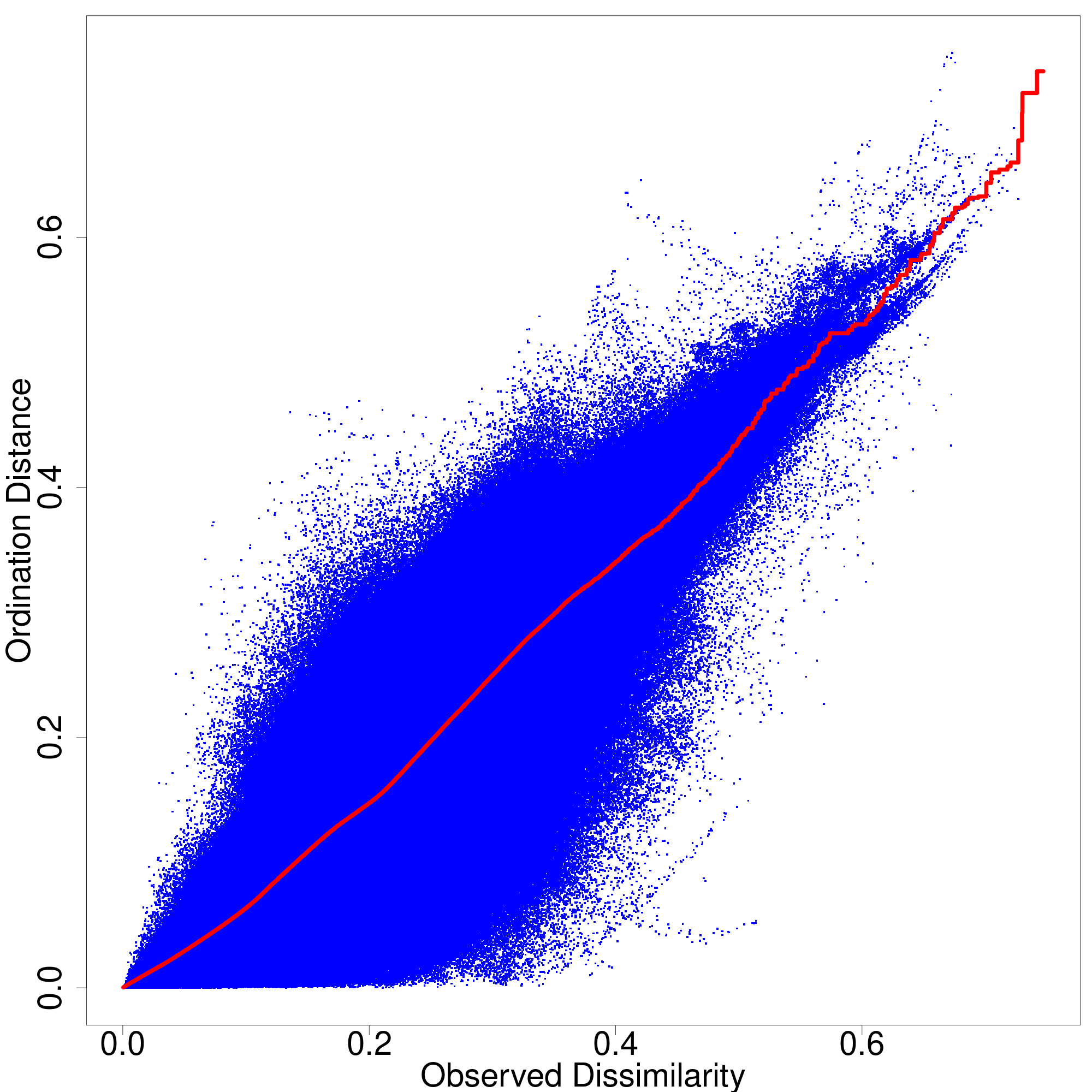
Figure S1.2. Shepard diagram of the NMDS


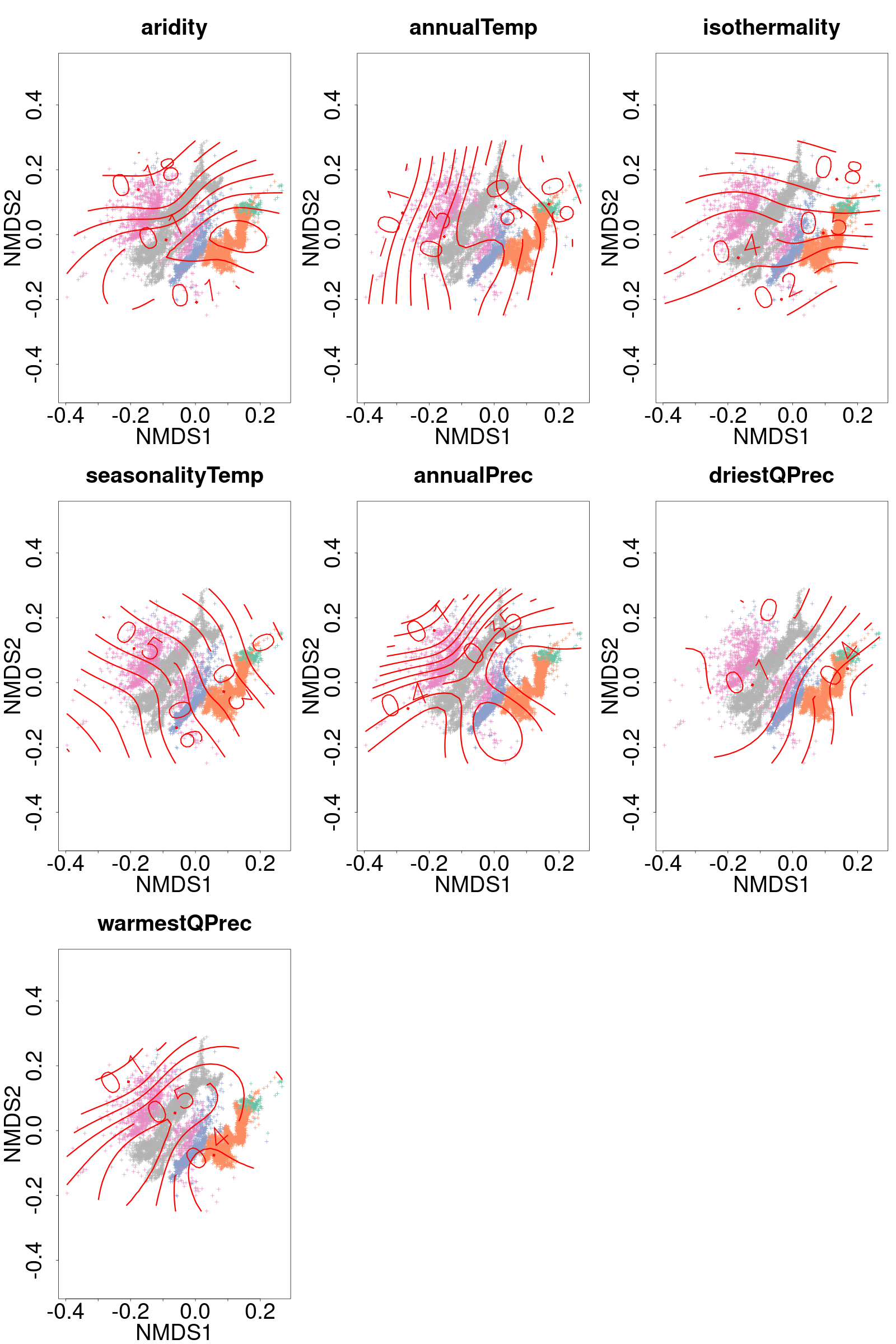
Figure S1.3. Trend surface of the macroclimatic variables fitted to the NMDS ordination space. Values on the contour lines are standardized and serve to check the direction and linear nature of the variable in the ordination space. Abbreviation of the macroclimatic variables are as follows: driestQPrec – precipitation of driest quarter; annualPrec – annual precipitation; annualTemp – annual mean temperature; warmestQPrec – precipitation of warmest quarter; seasonalityTemp – temperature seasonality. For color legend, please refer to Fig.S1.1.

## References

Bray, J. R. & Curtis, J. T. An Ordination of the Upland Forest Communities of Southern Wisconsin. *Ecol. Monogr.* **27**, 325–349. https://doi.org/10.2307/1942268 (1957).

Kruskal, J. B. Multidimensional scaling by optimizing goodness of fit to a nonmetric hypothesis. *Psychometrika* **29**, 1–27. https://doi.org/10.1007/BF02289565 (1964).
