# Supplementary material for: Empirical delineation of the forest-steppe zone is supported by macroclimate
Source: Sci Rep. 2023 Oct 13;13:17379. doi: 10.1038/s41598-023-44221-4 (PMC10575856; doi:10.1038/s41598-023-44221-4)
Supplement: Supplementary file 2 — Supplementary Information S2. [file 41598_2023_44221_MOESM2_ESM.docx]

# Appendix S2 – Details of the predictive distribution modeling

Ákos Bede-Fazekas et al.: Empirical delineation of the forest-steppe zone is supported by macroclimate. Scientific Reports

Boosted Regression Trees (BRT; a.k.a. gradient boosting model, GBM; Friedman et al. 2000, Friedman 2002, Schapire 2003) were used with fixed bag fraction (50%) and tree complexity (3). The learning rate was iteratively selected following the guideline of Elith et al. (2008). BRT is a flexible and robust predictive distribution modeling method with excellent predictive power (Elith et al. 2006, Bühlmann and Hothorn 2007, Velásquez-Tibatá et al. 2016). For the six studied distributions, i.e. that of the forest-steppe zone (studied by the 'zone' model) and its five regions (studied by the 'region' models), independent models were trained and later used to predict the probability of potential occurrence of the zone and its regions. Initial models were simplified with backward variable selection. For each model, observed occurrence data were split to 50-50% training and evaluation datasets stratified for prevalence using random spatial blocks with 250-km-long edges (Valavi et al. 2019). The relative importance for prediction (i.e. variable contribution) was estimated by the formula of Friedman (2001) that is based on the number of times a variable was selected for splitting, weighted by the squared improvement to the model as a result of each split, averaged over all trees, and, finally, rescaled so that the sum of all variable contributions adds to 100 (Friedman and Meulman 2003, Elith et al. 2008).

The trained models were evaluated by calculating the Area Under the ROC Curve (AUC; Hanley and McNeil 1982), which is one of the most widespread goodness-of-fit measures of predictive distribution models, on the evaluation dataset. To study whether macroclimate drives the distributional patterns or macroclimate is only a proxy for an underlying spatial structure that drives both climate and the distribution of forest-steppes, each model was repeated after replacing the macroclimatic variables with five coordinate-related variables (longitude, latitude, squared longitude, squared latitude, longitude × latitude), and AUC values were compared.

Predictions of the probability of potential occurrence, falling within the [0; 1] interval, were rescaled to a five-level ordinal scale (from 'not probable' to 'highly probable') using specific thresholds that account for observed presences (Somodi et al. 2017). Rescaling enhances interpretability and allows comparability of the predictions (Somodi et al. 2017). To capture potential inaccuracies, omission error (i.e. underprediction by the predictive distribution model or overestimation of the real distribution during delimitation) and commission error (i.e. overprediction by the predictive distribution model or underestimation of the real distribution during delimitation) were calculated as difference of the ordinal prediction and the presence (5) or absence (1). In each geographical point, both the omission and the commission errors were aggregated for the five region models, using their maximum value.

The predictive distribution model of the forest-steppe zone ('zone' model) trained 9850 trees with a learning rate of 0.1 (Table S2.1). The number of trees the predictive distribution models built varied between 3650–9500 when the presence/absence of the different regions were used for training ('region' models). The 'region' predictive distribution models used a similar or slower learning process than the 'zone' model (learning rates varied between 0.01 and 0.1).

Table S2.1. The used learning rate and the number of trees built by the 'zone' predictive distribution model and the five 'region' models.

| **model** | **learning rate** | **number of trees** |
| --- | --- | --- |
| 'zone' | 0.1 | 9850 |
| Southeast Europe | 0.01 | 7200 |
| East Europe | 0.1 | 3650 |
| West Siberia | 0.01 | 9500 |
| Inner Asia | 0.05 | 9400 |
| Far East | 0.05 | 7250 |

## References

Bühlmann, P. & Hothorn, T. Boosting algorithms: regularization, prediction and model fitting. *Stat. Sci.* **22**, 477–505. https://doi.org/10.1214/07-STS242 (2007)

Elith, J. *et al.* Novel methods improve prediction of species’ distributions from occurrence data. *Ecography* **29**, 129–151. https://doi.org/10.1111/j.2006.0906-7590.04596.x (2006).

Elith, J., Leathwick, J. R. & Hastie, T. A working guide to boosted regression trees. *J. Anim. Ecol.* **77**, 802–813. https://doi.org/10.1111/j.1365-2656.2008.01390.x (2008).

Friedman, J. H. Greedy function approximation: a gradient boosting machine. *Ann. Stat.* **29**, 1189–1232. https://doi.org/10.1214/aos/1013203451 (2001)

Friedman, J. H. Stochastic gradient boosting. *Computat. Stat. Data An.* **38**, 367–378. https://doi.org/10.1016/S0167-9473(01)00065-2 (2002).

Friedman, J. H., Hastie, T. & Tibshirani, R. Additive logistic regression: a statistical view of boosting. *Ann. Stat.* **28**, 337–407. https://doi.org/10.1214/aos/1016218223 (2000).

Friedman, J. H. & Meulman, J. J. Multiple additive regression trees with application in epidemiology. *Stat. Med.* **22**, 1365–1381. https://doi.org/10.1002/sim.1501 (2003)

Hanley, J. A. & McNeil, B. J. The meaning and use of the area under a receiver operating characteristic (ROC) curve. *Radiology* **143**, 29–36. https://doi.org/10.1148/radiology.143.1.7063747 (1982).

Schapire, R. The boosting approach to machine learning – an overview in *MSRI Workshop on Nonlinear Estimation and Classification* (eds. Denison, D. D. *et al.*) (Mathematical Sciences Research Institute, 2003).

Somodi, I. *et al.* Implementation and application of multiple potential natural vegetation models - a case study of Hungary. *J. Veg. Sci.* **28**, 1260–1269. https://doi.org/10.1111/jvs.12564 (2017).

Valavi, R., Elith, J., Lahoz-Monfort, J. J. & Guillera-Arroita, G. blockCV: An R package for generating spatially or environmentally separated folds for k-fold cross-validation of species distribution models. *Methods Ecol. Evol.* **10**, 225–232. https://doi.org/10.1111/2041-210X.13107 (2019).

Velásquez-Tibatá, J., Graham, C. H., Munch, S. B. Using measurement error models to account for georeferencing error in species distribution models. *Ecography* **39**, 305–316. https://doi.org/10.1111/ecog.01205 (2016).
