# Supplementary material for: Empirical delineation of the forest-steppe zone is supported by macroclimate
Source: Sci Rep. 2023 Oct 13;13:17379. doi: 10.1038/s41598-023-44221-4 (PMC10575856; doi:10.1038/s41598-023-44221-4)
Supplement: Supplementary file 3 — Supplementary Information S3. [file 41598_2023_44221_MOESM3_ESM.docx]

# Appendix S3 – Predictions with the 'region' models

Ákos Bede-Fazekas et al.: Empirical delineation of the forest-steppe zone is supported by macroclimate. Scientific Reports


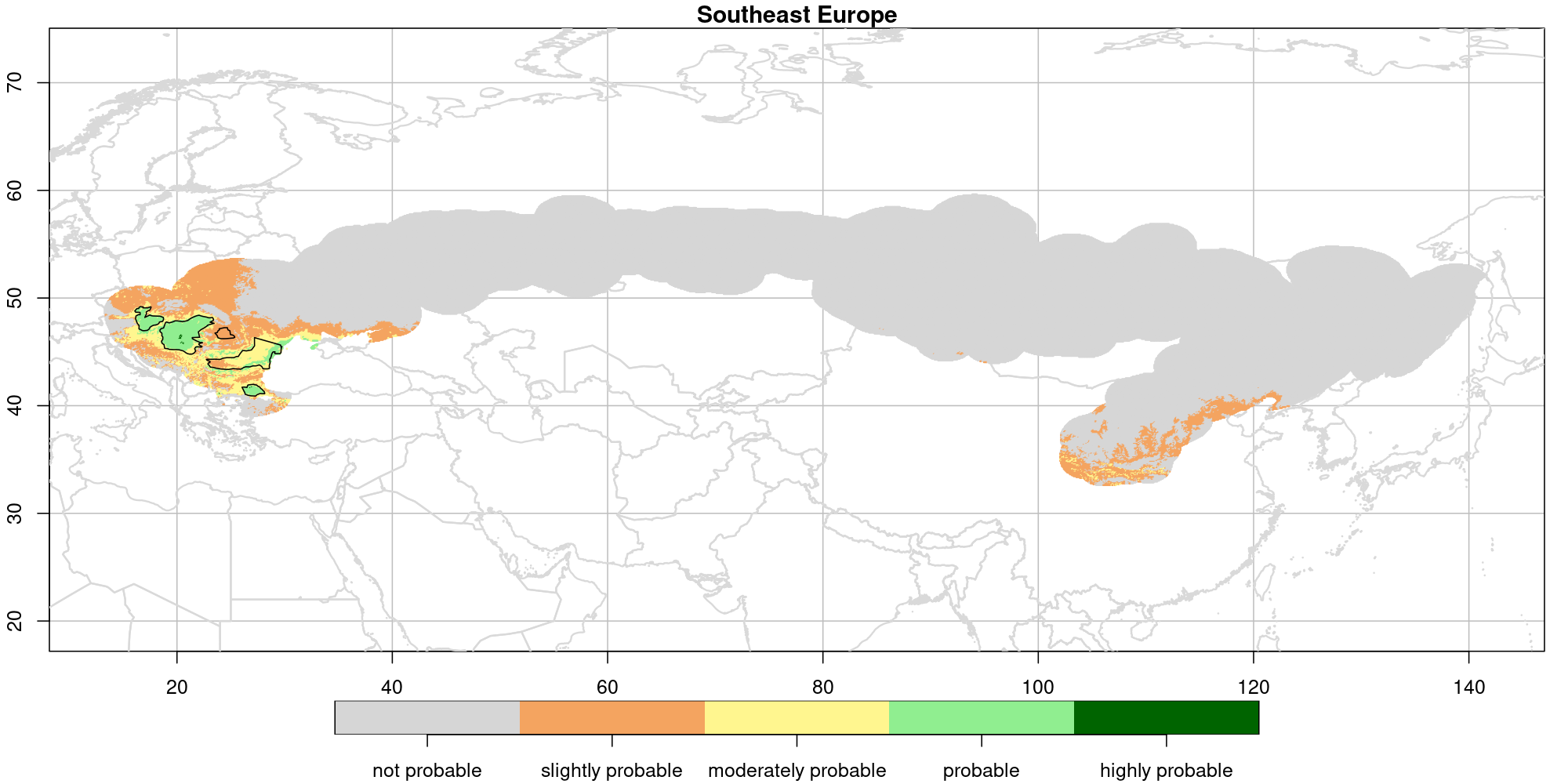
Figure S3.1. Predicted probability of occurrence of the Southeast Europe region according to the predictive distribution model. The distribution delineated by Erdős et al. (2018) is displayed with a solid black line.


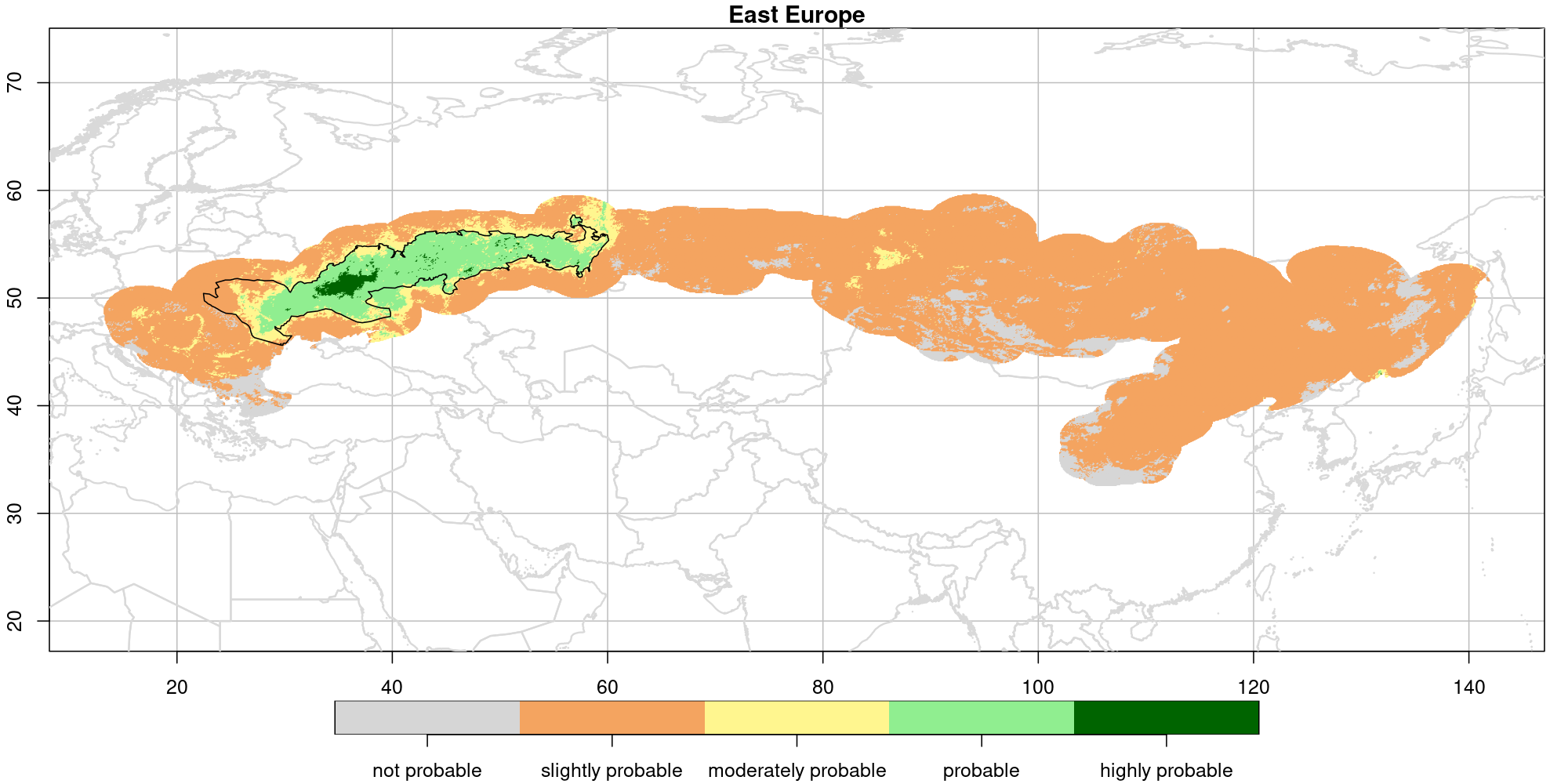
Figure S3.2. Predicted probability of occurrence of the East Europe region according to the predictive distribution model. The distribution delineated by Erdős et al. (2018) is displayed with a solid black line.


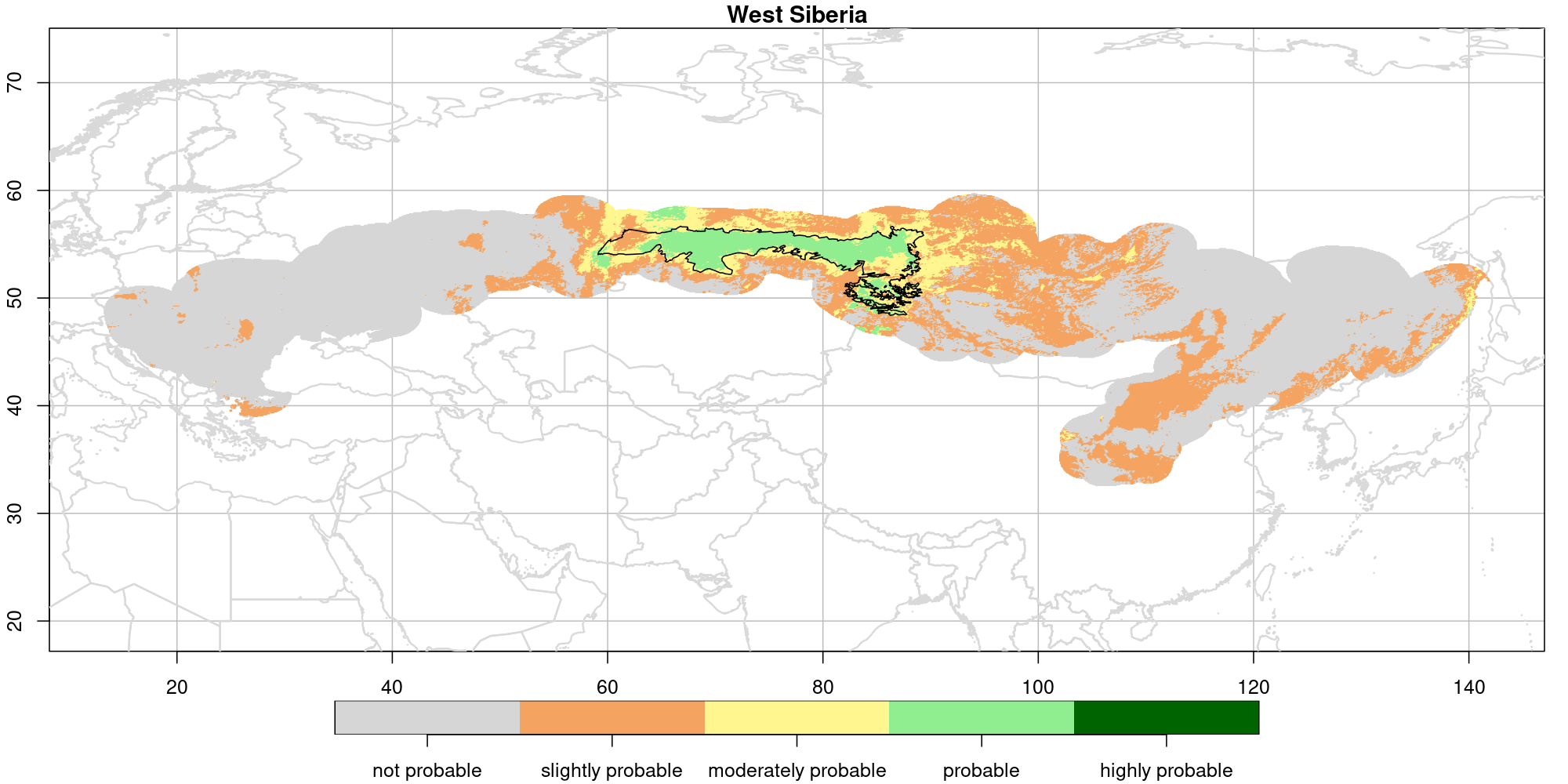
Figure S3.3. Predicted probability of occurrence of the West Siberia region according to the predictive distribution model. The distribution delineated by Erdős et al. (2018) is displayed with a solid black line.


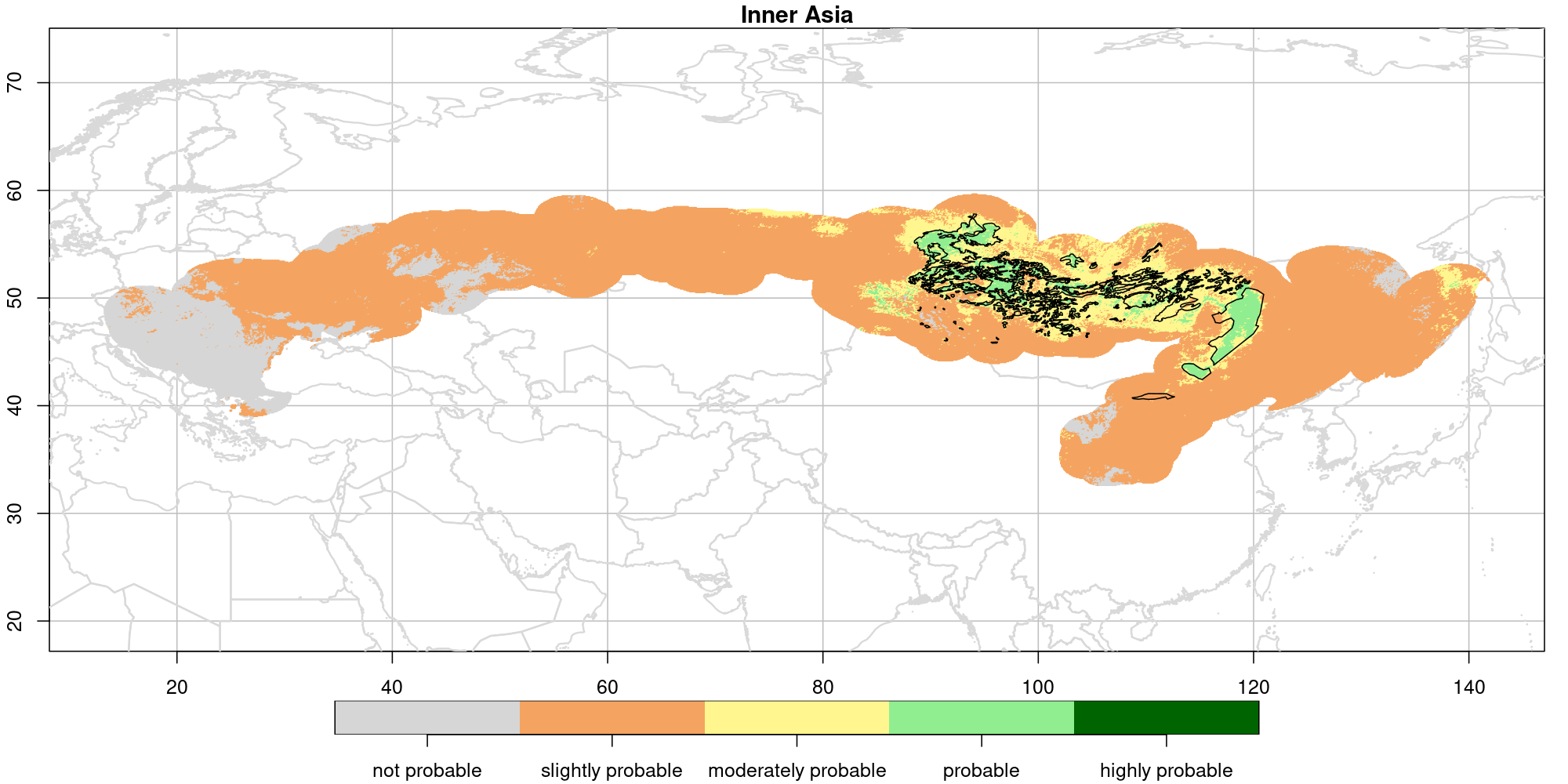
Figure S3.4. Predicted probability of occurrence of the Inner Asia region according to the predictive distribution model. The distribution delineated by Erdős et al. (2018) is displayed with a solid black line.


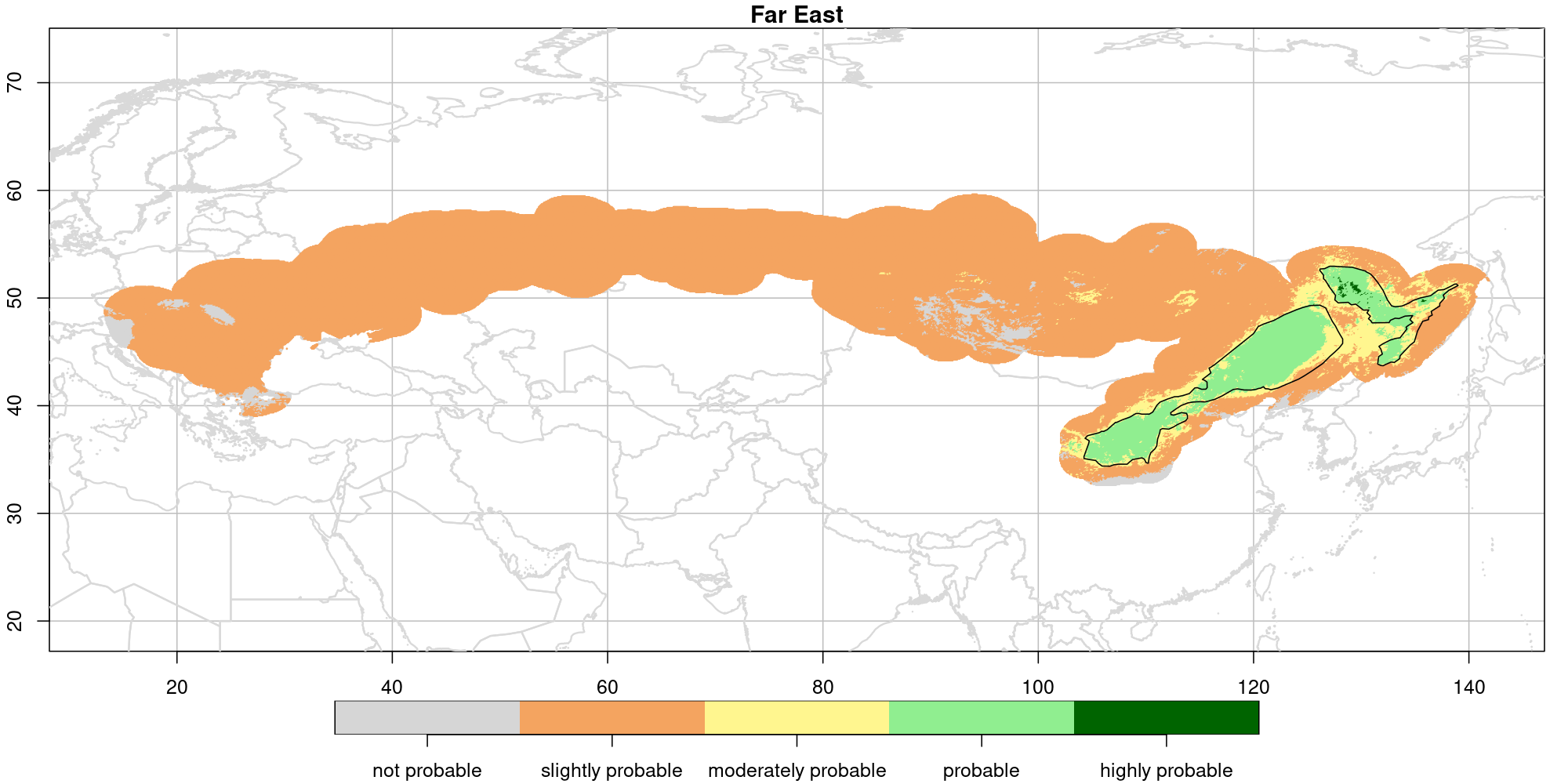
Figure S3.5. Predicted probability of occurrence of the Far East region according to the predictive distribution model. The distribution delineated by Erdős et al. (2018) is displayed with a solid black line.

## References

Erdős, L. *et al.* The edge of two worlds: A new review and synthesis on Eurasian forest-steppes. *Appl. Veg. Sci.* **21**, 345-362. https://doi.org/10.1111/avsc.12382 (2018).
