# Supplementary material for: Empirical delineation of the forest-steppe zone is supported by macroclimate
Source: Sci Rep. 2023 Oct 13;13:17379. doi: 10.1038/s41598-023-44221-4 (PMC10575856; doi:10.1038/s41598-023-44221-4)
Supplement: Supplementary file 4 — Supplementary Information S4. [file 41598_2023_44221_MOESM4_ESM.docx]

# Appendix S4 – Details of the forest-steppe map of Erdős et al. (2018)

When delineating Eurasian forest-steppes, Erdős et al. (2018) relied on the following maps: Keller (1927), Wilhelmy (1943), Leimbach (1948), Berg (1958), Horvat et al. (1974), Mayer (1984), Noirfalise (1987), Walter and Breckle (1989), Lavrenko and Karamysheva (1993), Zhu (1993), Wallis de Vries et al. (1996), Pócs (2000), Varga et al. (2000), Olson et al. (2001), Chibilyov (2002), Zlotin (2002), Bohn et al. (2004), Dulamsuren et al. (2005), Zólyomi (2007), Chytrý et al. (2008), Karácsonyi (2009), Baas et al. (2012), Chytrý (2012), Rachkovskaya and Bragina (2012), Smelansky and Tishkov (2012), Sanjmyatav (2012), Liu et al. (2015), Raingruber (2016), Wesche et al. (2016), Makunina (2017). All the above maps were digitized manually in ArcView GIS 3.2 (ESRI). When two or more maps were inconsistent, Erdős et al. (2018) generally opted for the wider concept, but the delineation also relied on expert knowledge.

## References

Baas, S., Tessitore, S. & Jellye, T. Rethinking pastoral risk management in Mongolia in *Eurasian steppes. Ecological problems and livelihoods in a changing world* (eds Werger, M. J. A. & van Staalduinen, M. A.) 507–546 (Springer, 2012).

Berg, L. S. Die geographischen Zonen der Sowjetunion I-II. (Teubner, 1958).

Bohn, U. *et al.* (eds.). Karte der natürlichen Vegetation Europas (Bundesamt für Naturschutz, 2004).

Chibilyov, A. Steppe and forest-steppe in *The physical geography of northern Eurasia* (ed. Shahgedanova, M.) 248–266 (Oxford University Press, 2002).

Chytrý, M. Vegetation of the Czech Republic: Diversity, ecology, history and dynamics. *Preslia* **84**, 427–504. (2012).

Chytrý, M. *et al.* Diversity of forest vegetation across a strong gradient of climatic continentality: Western Sayan Mountains, southern Siberia. *Plant. Ecol.* **196**, 61–83. https://doi.org/10.1007/s11258-007-9335-4 (2008).

Dulamsuren, C., Welk, E., Jäger, E. J., Hauck, M. & Mühlenberg, M. Range-habitat relationships of vascular plant species at the taiga forest steppe borderline in the western Khentey Mountains, northern Mongolia. *Flora* **200**, 376–397. https://doi.org/10.1016/j.flora.2005.02.002 (2005).

Erdős, L. *et al.* The edge of two worlds: A new review and synthesis on Eurasian forest-steppes. *Appl. Veg. Sci.* **21**, 345–362. https://doi.org/10.1111/avsc.12382 (2018).

Horvat, I., Glavač, V. & Ellenberg, H. Vegetation Südosteuropas. (Gustav Fischer, 1974).

Karácsonyi, D. Ein Versuch der Typologie der ländlichen Räume in der Ukraine. *Eur. Region.* **17**, 34–50. (2009).

Keller, B. A. Distribution of vegetation on the plains of European Russia. *J. Ecol.* **15**, 189–233. https://doi.org/10.2307/2255990 (1927).

Lavrenko, E. M. & Karamysheva, Z. V. Steppes of the former Soviet Union and Mongolia in *Ecosystems of the world 8B. Natural grasslands. Eastern hemisphere and résumé* (ed. Coupland, R. T.) 3–59 (Elsevier, 1993).

Leimbach, W. Zur Waldsteppenfrage in der Sowjetunion. *Erdkunde* **2**, 238–256. (1948)

Liu, H., Yin, Y., Wang, Q. & He, S. Climatic effects on plant species distribution within the forest-steppe ecotone in northern China. *Appl. Veg. Sci.* **18**, 43–49. https://doi.org/10.1111/avsc.12139 (2015).

Makunina, N. I. Biodiversity of basic vegetation communities in forest steppes of the Altai-Sayan mountain region. *Int. J. Environ. Stud.* **74**, 674–684. https://doi.org/10.1080/00207233.2017.1283943 (2017).

Mayer, H. Wälder Europas (Gustav Fischer Verlag, 1984).

Noirfalise, A. Map of the natural vegetation of the member countries of the European Community and the Council of Europe. 2^nd^ ed. (Office for Official Publications of the European Communities, 1987).

Olson, D. M. *et al*. Terrestrial ecoregions of the world: a new map of life on Earth. *Bioscience.* **51**, 933–938. https://doi.org/10.1641/0006-3568(2001)051[0933:TEOTWA]2.0.CO;2 (2001)

Pócs, T. Növényföldrajz in *Növényföldrajz, társulástan és ökológia* (eds. Hortobágyi, T. & Simon, T.) 25–166 (Nemzeti Tankönyvkiadó, 2000).

Rachkovskaya, E. I. & Bragina, T. M. Steppes of Kazakhstan: Diversity and present state in *Eurasian steppes. Ecological problems and livelihoods in a changing world* (eds. Werger, M. J. A. & van Staalduinen, M. A.) 103–148 (Springer, 2012).

Raingruber, A. A network of the steppe and forest steppe along the Prut and Lower Danube rivers during the 6th millennium BC. *Documenta Praehistorica* **43**, 167–181. https://doi.org/10.4312/dp.43.8 (2016).

Sanjmyatav, T. Mongolian nomads and climate change – A herder’s view in *Eurasian steppes. Ecological problems and livelihoods in a changing world* (eds. Werger M. J. A. & van Staalduinen, M. A.) 547–559 (Springer, 2012).

Smelansky, I. E. & Tishkov, A. A. The steppe biome in Russia: Ecosystem services, conservation status, and actual challenges in *Eurasian steppes. Ecological problems and livelihoods in a changing world* (eds. Werger M. J. A. & van Staalduinen, M. A.) 45–101 (Springer, 2012).

Varga, Z. *et al.* Az erdőssztyepp fogalma, típusai és jellemzésük in *Alföldi erdőssztyepp-maradványok Magyarországon* (eds. Molnár, Z. & Kun, A.) 7–19 (WWF-MTA ÖBKI, 2000).

Wallis de Vries, M. F., Manibazar, N. & Dügerlham, S. The Vegetation of the Forest-Steppe Region of Hustain Nuruu, Mongolia. *Vegetatio* **122**, 111–127. https://doi.org/10.1007/BF00044694 (1996).

Walter, H. & Breckle, S-W. Ecological systems of the geobiosphere 3 – Temperate and polar zonobiomes of northern Eurasia (Springer, 1989).

Wesche, K. *et al.* The Palaearctic steppe biome: A new synthesis. *Biodivers. Conserv.* **25**, 2197–2231. https://doi.org/10.1007/s10531-016-1214-7 (2016).

Wilhelmy, H. Das Wald-, Waldsteppen- und Steppenproblem in Südrußland. *Geographische Zeitschrift* 49, 161–188. (1943).

Zhu, T-C. Grasslands of China in *Ecosystems of the world 8B. Natural grasslands. Eastern hemisphere and résumé* (ed. Coupland, R. T.) 61-82 (Elsevier, 1993).

Zlotin, R. Biodiversity and productivity of ecosystems in *The physical geography of northern Eurasia* (ed. Shahgedanova, M.) 169–190. (Oxford University Press, 2002).

Zólyomi, B. Magyarország természetes növénytakarója in *Pannon enciklopédia: Magyarország növényvilága* (ed. Járai-Komlódi, M.) 156–157 (Urbis, 2007).
