# Supplementary figures and images for: Empirical delineation of the forest-steppe zone is supported by macroclimate
Source: Sci Rep. 2023 Oct 13;13:17379. doi: 10.1038/s41598-023-44221-4 (PMC10575856; doi:10.1038/s41598-023-44221-4)

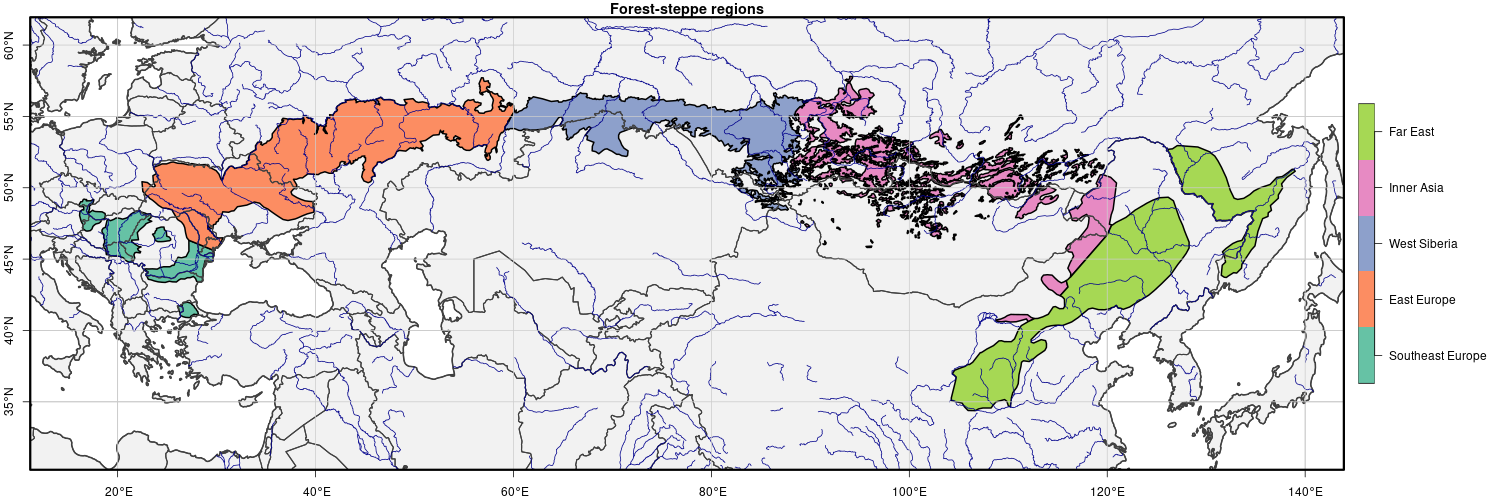

Supplement: Supplementary file 5 — Supplementary Information S5. [file 41598_2023_44221_MOESM5_ESM.zip › forest_steppe.png]

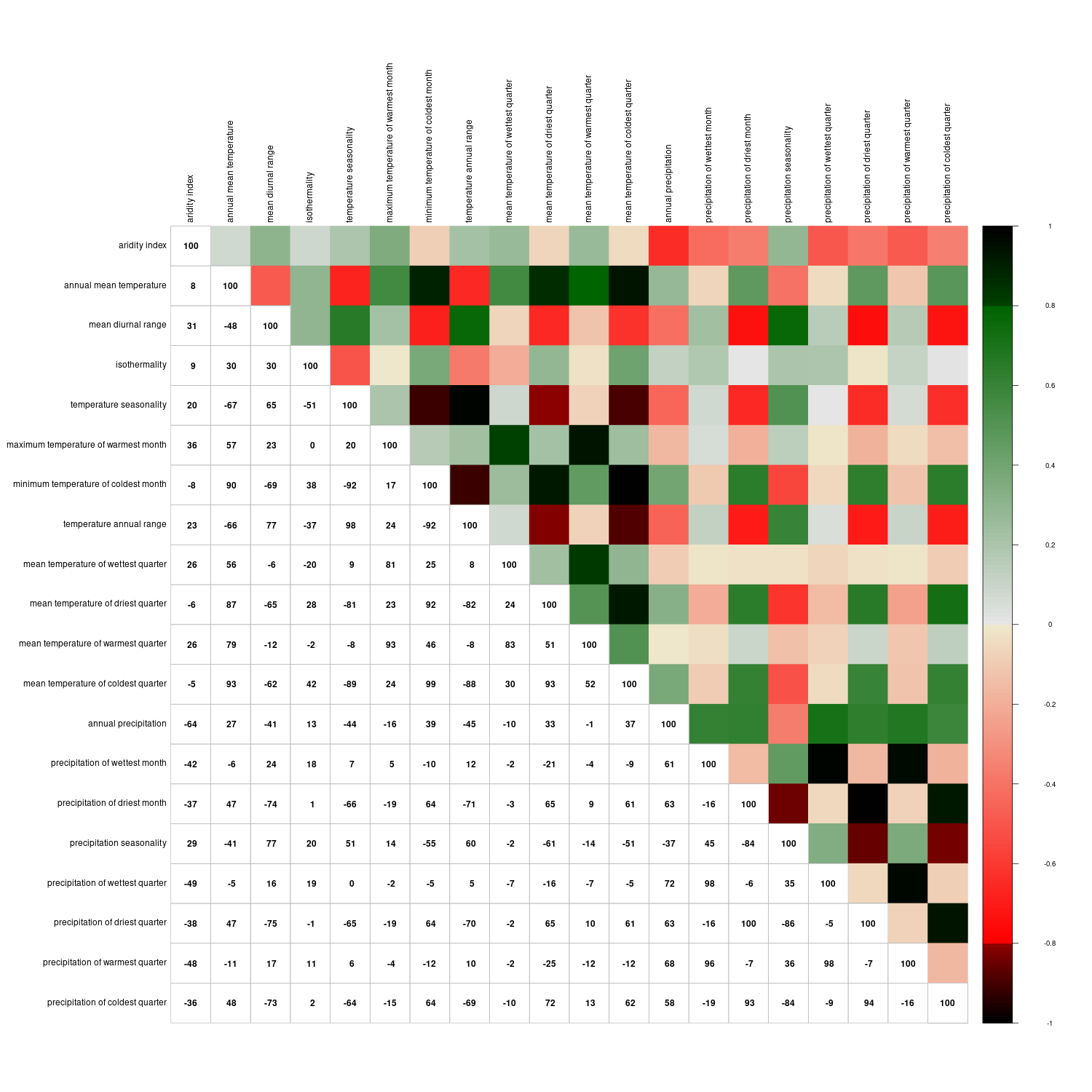

Supplement: Supplementary file 7 — Supplementary Information S7. [file 41598_2023_44221_MOESM7_ESM.zip › correlation_matrix.png]
