# Supplementary material for: Empirical delineation of the forest-steppe zone is supported by macroclimate
Source: Sci Rep. 2023 Oct 13;13:17379. doi: 10.1038/s41598-023-44221-4 (PMC10575856; doi:10.1038/s41598-023-44221-4)
Supplement: Supplementary file 6 — Supplementary Information S6. [file 41598_2023_44221_MOESM6_ESM.docx]

# Appendix S6 – List of bioclimatic variables

Ákos Bede-Fazekas et al.: Empirical delineation of the forest-steppe zone is supported by macroclimate. Scientific Reports

Table S6.1. List of bioclimatic variables available from the WorldClim database (Fick and Hijmans 2017) and whether these were used as predictors in the 'zone' and 'region' models

| **Full name** | **Predictor in the 'zone' model** | **Predictor in the 'region' models** |
| --- | --- | --- |
| annual mean temperature | × | × |
| mean diurnal range |  |  |
| isothermality |  | × |
| temperature seasonality | × | × |
| maximum temperature of warmest month |  |  |
| minimum temperature of coldest month |  |  |
| temperature annual range |  |  |
| mean temperature of wettest quarter |  |  |
| mean temperature of driest quarter |  |  |
| mean temperature of warmest quarter | × |  |
| mean temperature of coldest quarter |  |  |
| annual precipitation | × | × |
| precipitation of wettest month |  |  |
| precipitation of driest month |  |  |
| precipitation seasonality | × | × |
| precipitation of wettest quarter |  |  |
| precipitation of driest quarter | × | × |
| precipitation of warmest quarter | × | × |
| precipitation of coldest quarter |  |  |

## References

Fick, S. E. & Hijmans, R. J. WorldClim 2: new 1-km spatial resolution climate surfaces for global land areas. *Int. J. Climatol.* **37**, 4302–4315. http://dx.doi.org/10.1002/joc.5086 (2017).
